# Differences in Access to and Preferences for Using Patient Portals and Other eHealth Technologies Based on Race, Ethnicity, and Age: A Database and Survey Study of Seniors in a Large Health Plan

**DOI:** 10.2196/jmir.5105

**Published:** 2016-03-04

**Authors:** Nancy P Gordon, Mark C Hornbrook

**Affiliations:** ^1^ Kaiser Permanente Northern California Division of Research Oakland, CA United States; ^2^ Kaiser Permanente Northwest Region The Center for Health Research Portland, OR United States

**Keywords:** eHealth disparities, patient portals, race-ethnic disparities, seniors, Internet use, health information technology disparities

## Abstract

**Background:**

Patients are being encouraged to go online to obtain health information and interact with their health care systems. However, a 2014 survey found that less than 60% of American adults aged 65 and older use the Internet, with much lower usage among black and Latino seniors compared with non-Hispanic white seniors, and among older versus younger seniors.

**Objective:**

Our aims were to (1) identify race/ethnic and age cohort disparities among seniors in use of the health plan’s patient portal, (2) determine whether race/ethnic and age cohort disparities exist in access to digital devices and preferences for using email- and Web-based modalities to interact with the health care system, (3) assess whether observed disparities in preferences and patient portal use are due simply to barriers to access and inability to use the Internet, and (4) learn whether older adults not currently using the health plan’s patient portal or website have a potential interest in doing so in the future and what kind of support might be best suited to help them.

**Methods:**

We conducted two studies of seniors aged 65-79 years. First, we used administrative data about patient portal account status and utilization in 2013 for a large cohort of English-speaking non-Hispanic white (n=183,565), black (n=16,898), Latino (n=12,409), Filipino (n=11,896), and Chinese (n=6314) members of the Kaiser Permanente Northern California health plan. Second, we used data from a mailed survey conducted in 2013-2014 with a stratified random sample of this population (final sample: 849 non-Hispanic white, 567 black, 653 Latino, 219 Filipino, and 314 Chinese). These data were used to examine race/ethnic and age disparities in patient portal use and readiness and preferences for using digital communication for health-related purposes.

**Results:**

Adults aged 70-74 and 75-79 were significantly less likely than 65-69 year olds to be registered to use the patient portal, and among those registered, to have used the portal to send messages, view lab test results, or order prescription refills. Across all age groups, non-Hispanic whites and Chinese seniors were significantly more likely than black, Latino, and Filipino seniors to be registered and to have performed these actions. The survey found that black, Latino, and Filipino seniors and those 75 years old and older were significantly less likely to own digital devices (eg, computers, smartphones), use the Internet and email, and be able and willing to use digital technology to perform health care-related tasks, including obtaining health information, than non-Hispanic whites, Chinese, and younger seniors (aged 65-69), respectively. The preference for using non-digital modalities persisted even among Internet users.

**Conclusions:**

Health plans, government agencies, and other organizations that serve diverse groups of seniors should include social determinants such as race/ethnicity and age when monitoring trends in eHealth to ensure that eHealth disparities do not induce greater health status and health care disparities between more privileged and less privileged groups.

## Introduction

The adoption of digital technology has been accelerating rapidly, and the Internet has become an important tool for health care-related communications and transactions. Increasingly, health care organizations and government agencies are using their websites as key modes of informing patients and the public about health, health care, and health care coverage. In addition, email and secure website portals are used for informational, health care delivery, and business transactions [1]. This rapid shift to Web-based transactions among health care providers is in part being driven by Centers for Medicare and Medicaid Services (CMS) “Meaningful Use” requirements that mandate the deployment of digital technology to increase patient engagement with their health and health care outside the clinic setting [2,3], and in part by growing consumer demand for online access to health and health care information [4-8]. As health care organizations continue to embrace expanded uses of their websites and other health information (eHealth) technologies as primary channels for delivery of health and health care information, patient education, provider-patient communication, and health care-related business transactions, a worrisome health policy issue is exacerbation, rather than elimination, of health and health care disparities among already vulnerable populations if universal adoption of eHealth technologies does not occur [3,9,10].

It is well documented that digital divides exist in the general US population by race/ethnicity, income, educational attainment, and health literacy [11-31]. Recent studies have found similar disparities in use of patient portals [13,23-29,32,33] and the Internet as a source of health information [11-14,23,24,29]. Although older adults are among the fastest growing group of Internet users in the United States, surveys show that their use still significantly lags behind even that of middle-aged adults. In 2014, an estimated 87% of US adults used the Internet to access websites and/or to exchange emails [31]. In 2013, 59% of adults (72% of Internet users) had looked on the Internet for health information of some kind in the past year [6]. However, adults aged ≥65 years were significantly less likely than those aged 50-64 to be Internet users (57% vs 88%, respectively) [31] and significantly less likely to have gone online for health information in the past year (33% of all seniors and 58% of senior Internet users vs 62% of 50-64 year olds and 71% of Internet users in that age group) [6]. Within the senior age group, computer access and ability to use the Internet has been shown to be lower among blacks and Hispanic/Latinos than among non-Hispanic whites [12,17,18,27,32,34-36], those aged ≥75 [12,16,17,31,34-39], those with a high school diploma or less [12,17,18,26,34-36], those with a low household income [12,18,35], and those with low levels of literacy and health literacy [32,38].

To date, limited information has been available about the extent to which race/ethnic and age-related eHealth digital divides exist within the senior age group, and beyond access issues, are a function of eHealth literacy and preferences for using digital technology for health-related purposes. Additionally, of the relatively few studies that have focused on race/ethnic differences among seniors, most have been restricted to non-Hispanic whites, African-American/blacks, and Hispanic/Latinos, leaving a gap in information regarding use of digital technology for health-related purposes among the growing Asian segment of the senior population.

Seniors are being expected to make the shift from print and telephonic health communications to interacting via websites, email, text messages, and interactive voice response systems along with other adult age groups. As such, an emerging research and policy priority is to identify the extent to which age and race/ethnic differences in seniors’ access to and comfort with using eHealth have the potential to create or exacerbate disparities in access to timely health care–related information, patient education, and lower-cost health care options such as video visits and online ordering and purchasing of prescription medications and medical equipment. Recognizing this potential, *Healthy People 2020* included an expanded set of goals for use of “health communication strategies and health information technology to improve population health outcomes and health care quality and to achieve health equity” [40].

As part of “Stage 3 Meaningful Use” requirements for electronic medical record systems, health plans, hospitals, and medical offices may be asked to identify and act on patient communication preferences for clinical summaries, reminders, and patient educational materials [41]. From a health care provider perspective, this generates an imperative to understand how the characteristics of Medicare-age members may affect meeting meaningful use targets. Member engagement with a health plan’s portal and/or website may be more limited for plans with a high percentage of older members who cannot or prefer not to go online for health care transactions. Similarly, government and non-governmental agencies and organizations that serve seniors should take into account the health care-related digital divide when developing information technology (IT) programs, planning for dissemination of important information, and requiring information and communications to be transmitted online.

In this study, we assessed the extent to which race/ethnic and age-related eHealth digital divides exist among the racially and ethnically diverse seniors of Kaiser Permanente Northern California (KPNC) and what might be driving the divides that are observed. We used a two-pronged approach. We first examined race/ethnic and age-group differences in overall registration to use and patterns of use of four features of the health plan’s secure patient portal in 2013 in a large study population of non-Hispanic white, black, Hispanic/Latino, Filipino, and Chinese adults aged 65-79. Concurrently, we surveyed a sample of this population to obtain information about the types of digital devices (eg, computer, mobile phone, tablet) and digital technologies (Internet, email, text messaging, Skype) they were using, as well as their readiness and preferences for using digital modalities for health-related purposes. The study had four main aims: (1) to identify race/ethnic and age cohort disparities among seniors in use of the health plan’s patient portal, (2) to determine whether race/ethnic and age cohort disparities exist in access to digital devices and preferences for using email- and Web-based modalities to interact with the health care system, (3) to assess whether observed disparities in preferences and patient portal use are due simply to barriers to access and inability to use the Internet, and (4) to learn whether older adults who are not currently using the health plan’s patient portal or website have a potential interest in doing so in the future and, if so, what kind of support might be best suited to help them.

## Methods

### Setting

KPNC is a vertically integrated health care delivery system that serves over 2.4 million adult members and their family members who mostly reside or work in the San Francisco Bay Area, Silicon Valley, Sacramento area, or the Central Valley in Northern California. The KPNC adult membership is highly similar to the insured population of Northern California with regard to demographic and health characteristics [42]. KPNC has a comprehensive website that provides health plan and health information (eg, about health conditions, medications, healthy behaviors/lifestyle) accessible to both members and the general public, and a secure patient portal that is available only to health plan members who register for and activate a patient portal account. Once members activate their account, they can use a variety of secure features on the website. These features include communicating with their health care providers and Member Services specialists using secure messaging, viewing laboratory test results, ordering and paying for prescription refills, viewing and scheduling appointments for primary care and vision care, checking their preventive care status (eg, use of recommended immunizations and cancer screening services) and their prescribed medication list, completing online health questionnaires, using patient/health education programs not available to the public, and downloading a variety of forms for use within Kaiser Permanente.

### Study Population

Our primary aim was to determine whether race/ethnic and age-related differences exist in preferences for using the health plan’s patient portal features and health education resources, which are primarily available in English. The health plan also has Spanish language websites, but at the time of this study, these did not have full functionality in Spanish and were not as comprehensive with regard to health information. Because previous research has shown a sharp drop in Internet use after age 75, we restricted the study population to members aged 65-79 who had no indication in health plan records of having a preference for oral or written communication in a language other than English (non–limited English proficient [non-LEP]). Within this age group, we restricted our study to a cohort of adults who had been assigned to one of the health plan’s five largest race/ethnic groups: non-Hispanic white, African-American/black (black), Hispanic/Latino (Latino), Filipino-American (Filipino), or Chinese-American (Chinese) using data from administrative and research sources. In 2013, these five race/ethnic groups accounted for approximately 95% of all non-LEP health plan members aged ≥65. Furthermore, members aged 65-79 in these race/ethnic groups accounted for approximately 75% of all non-LEP members aged ≥65. Because we wanted everyone to have had at least 2 years of opportunity and encouragement to create a kp.org account and to use the website’s secure features, we further restricted the study population to people who in November 2013 had been continuous KPNC members for at least 2.5 years.

### Patient Portal Use Study

The full study population for the patient portal use study included 183,565 non-Hispanic white members, 16,898 black members, 12,409 Latino members, 11,896 Filipino members, and 6314 Chinese members aged 65-79. Of these, 114,752 non-Hispanic white, 13,006 black, 8755 Latino, 9329 Filipino, and 4087 Chinese members were in the health plan’s diabetes, hypertension, and/or coronary artery disease registry. We used the full study sample to calculate percentages of members who were registered to use the kp.org patient portal and from whom at least one secure email had been received by December 31, 2013. We used the subgroup of members who had at least one laboratory test in the 2013 calendar year to calculate percentages who viewed lab test results online at least once in 2013, and the subgroup who had at least one prescription refill in 2013 to calculate percentages who used the online prescription refill ordering feature at least once. We also calculated use of these secure features, plus signing into the secure portal at least once during the calendar year, among the same subgroups of members, first restricting analyses to those who had a kp.org account by the end of 2013 and then restricting to those in a chronic disease registry. It should be noted that these members may not have had a kp.org account or activated a kp.org account at the time they might have wanted to communicate with a doctor, obtain a lab test result, or order a prescription refill. However, in 2013, it was possible for nearly all adult members to create, activate, and immediately start to use a kp.org account within a few minutes.

All analyses for the patient portal study were conducted using SAS version 9.3 [43]. Proc Means was used to generate percentages, and multivariable models run using Proc Logistic assessed whether registration and use significantly (*P*<.001) differed across age groups (70-74 and 75-79 vs 65-69), race/ethnic groups (black, Latino, Filipino, and Chinese vs non-Hispanic white) for ages 65-79 and individual age groups, and age groups within each race or ethnicity. Denominators for Tables 1 and 2 cell percentages are provided in Multimedia Appendix 1.

### Survey Study

#### Sampling Design

From the study population, we selected stratified random samples of approximately equal numbers of women and men from three age groups (65-69, 70-74, 75-79) within each race/ethnic group: 1320 non-Hispanic whites, 1320 blacks, 1320 Latinos, 510 Filipinos, and 510 Chinese. The Filipino and Chinese samples were smaller than the others because their data were originally intended to be used for pilot study purposes.

#### Data Collection

The survey was conducted using a mailed print questionnaire available only in English, with interviewer administration upon request. An online option was not made available due to our prior experience that a very small percentage of seniors choose to participate using an online questionnaire when both modalities are offered. Participants were offered a US $5 gift card as recognition for returning a completed survey. The first survey was mailed in mid-November 2013, and a second mailing was conducted in mid-December 2013 to those who had not responded. People who did not respond to either of the first two survey mailings were sent a third, slightly shorter, questionnaire in early February 2014. Participants were told that the survey was being done to help Kaiser Permanente and other organizations learn about seniors’ use of digital tools (like computers, mobile devices, and the Internet) and how they prefer to give and get information about their health and health care. The survey materials stated that participation was important even if they did not use a computer, the Internet, email, or a mobile phone, or did not use the Kaiser Permanente website and did not want to use it. A copy of the survey questionnaire can be found in Multimedia Appendix 2.

#### Data Analysis

Survey respondents were assigned analytic weighting factors to adjust for sampling design and nonresponse. The weighting factors were created by dividing the number of people in the full study population who were in the age–sex–race/ethnicity–kp.org account status stratum that the respondent was representing by the number of survey respondents in that stratum. Patient portal account status was included as a component of the weighting after we discovered that in several race/ethnicity × age group strata, members who had signed up for a kp.org account by the time of the survey were significantly more likely to have responded than those who had not. Because approximately 6.84% (178/2602) of the sample completed the slightly shorter form of the survey, separate sets of weighting factors were created for those items included in both longer and shorter forms of the survey and for those items that were included only in the longer form. The raw (ie, unweighted) and weighted age-sex composition of the race/ethnic groups and the full sample are available on request.

All analyses were conducted using SAS version 9.3 procedures for complex datasets [43]. Proc Surveymeans was used to produce weighted percentages with 95% confidence intervals. Proc Surveylogistic was used to test whether statistically significant differences between age cohorts were observed (ie, 70-74 and 75-79 vs 65-69) for the full respondent sample, within race/ethnic groups, between race/ethnic groups (ie, black, Latino, Filipino, Chinese vs non-Hispanic white) for ages 65-79, and to test for significant differences by race/ethnicity, age, and other independent variables (eg, being an Internet user, being in fair or poor health, no formal education beyond high school) after controlling for multiple factors. All comparisons cited as statistically significant in the text had a Wald chi-square value of *P*<.05. No adjustment was made for multiple comparisons, but results of all comparisons are reported.

Kaiser Foundation Research Institute’s Institutional Review Board approved both the patient portal and survey studies.

## Results

### Patient Portal Use

In the full study population and across all racial and ethnic groups, older seniors (ie, adults aged 70-74 and 75-79) were significantly less likely than those aged 65-69 to have registered to use the patient portal, to have signed into the patient portal at least once, and to have used the patient portal to send a secure message, view lab test results online, or order prescription refills at least once by the end of the year (see Table 1). Across all age groups, black, Latino, and Filipino health plan members were significantly less likely than non-Hispanic white and Chinese members to have created a kp.org account by December 31, 2013, and to have used its secure patient portal features. Only 26.35% (1472/5587) of black members aged 75-79 years used the patient portal at least once in 2013 to send a message to their doctor, view a lab test result, refill a prescription, or make a doctor’s appointment, as compared to 56.31% (33,930/60,255) of non-Hispanic white members in the same age group. These race/ethnic and age group differences in the use of the patient portal were present even among members who had a kp.org account during at least part of 2013. Even among members included in one or more of the health plan’s chronic disease registries, significant age group and race/ethnic differences were observed in use of secure messaging and any of the four patient portal features (see Table 2).

### Characteristics of Survey Respondents

The overall survey response rate was 53.45% (2602/4868) after excluding ineligibles (14 not reachable by mail, 65 non-members, 32 deceased, 1 with dementia). Response rates were similar across age groups: 52.01% (841/1617) for ages 65-69, 53.87% (878/1630) for ages 70-74, and 54.47% (883/1621) for ages 75-79, with no significant sex difference within age group. However, response rates differed significantly by race/ethnic group: 65.26% (849/1301) for non-Hispanic whites, 44.44% (567/1276) for blacks, 50.50% (653/1293) for Latinos, 44.42% (219/493) for Filipinos, and 62.18% (314/505) for Chinese, with no significant differences in response by age and sex within each race/ethnic group.

**Table 1:** Registration for and use of the patient portal by age group and race/ethnicity^a^.

Use of the patient portal in 2013		Age	All	Non-Hispanic white	Black	Latino	Filipino	Chinese
**Was registered to use the patient portal by end of 2013, %**								
		65-79	77.1	81.1	54.1^b^	62.5^b^	60.5^b^	81.4
		65-69	82.2	86.3	61.3^b^	67.0^b^	65.4^b^	86.1
		70-74	78.6^c^	82.6^c^	55.4^b,c^	63.7^b,d^	61.1^b,c^	83.7
		75-79	71.5^c^	75.5^c^	47.3^b,c^	57.9^b,c^	55.2^b,c^	75.6^b,c^
**Signed onto the patient portal ≥1 time in 2013 (if registered to use patient portal at least part of 2013), %**								
		65-79	80.5	82.2	65.9^b^	70.8^b^	68.9^b^	85.5^b^
		65-69	83.3	85.0	69.4^b^	75.6^b^	74.1^b^	87.5
		70-74	81.6^c^	83.5^c^	66.9^b^	71.8^b,d^	69.1^b,c^	86.2^b^
		75-79	76.4^c^	78.2^c^	61.3^b,c^	65.7^b,c^	63.3^b,c^	83.1^b,c^
**Used the patient portal to send a message to a doctor, view lab test results, order a prescription refill, or make an appointment ≥1 time in 2013, %**								
	**All members**							
		65-79	59.5	64.2	32.9^b^	41.4^b^	38.8^b^	67.3^b^
		65-69	65.9	70.8	39.6^b^	47.7^b^	45.3^b^	72.7
		70-74	61.7^c^	66.6^c^	34.3^b,c^	42.8^b,c^	39.3^b,c^	70.1^b^
		75-79	51.9^c^	56.3^c^	26.3^b,c^	35.1^b,c^	32.3^b,c^	60.3^c,e^
	**Members registered to use the patient portal for at least part of 2013**							
		65-79	77.1	79.1	60.9^b^	66.2^b^	64.1^b^	82.6^b^
		65-69	80.1	82.0	64.7^b^	71.2^b^	69.3^b^	84.4
		70-74	78.5^c^	80.6 ^c^	62.0^b^	67.2^b,d^	64.3^b^	83.8^b^
		75-79	72.6^c^	74.6 ^c^	55.7^b,c^	60.7^b,c^	58.5^b,c^	79.8^b,c^
**Sent a secure message through the patient portal ≥1 time in 2013, %**								
	**All members**							
		65-79	46.3	50.8	23.3^b^	30.1^b^	26.1^b^	49.1
		65-69	52.3	56.9	29.4^b^	35.2^b^	31.9^b^	54.2
		70-74	48.1^c^	52.8^c^	24.1^b,c^	31.1^b,c^	26.5^b,c^	52.0
		75-79	39.7^c^	43.7^c^	17.9^b,c^	25.4^b,c^	20.4^b,c^	42.2^c^
	**Members registered to use the patient portal for at least part of 2013**							
		65-79	60.1	62.6	43.1^b^	48.2^b^	43.1^b^	60.3^b^
		65-69	63.5	66.0	47.9^b^	52.5^b^	48.8^b^	63.0
		70-74	61.3^b^	63.9^b^	43.6^b,c^	48.8^c^	43.3^b,c^	62.1
		75-79	55.5^b^	57.9^b^	37.9^b,c^	44.0^b,c^	36.9^b,c^	55.8^c^
**Viewed lab test results using the patient portal ≥1 time in 2013, %**								
	**All members who had ≥1 lab test in 2013**							
		65-79	62.8	68.0	33.9^b^	42.7^b^	40.0^b^	69.1
		65-69	69.2	74.5	40.5^b^	49.6^b^	47.8^b^	74.6
		70-74	64.6^c^	69.9^c^	35.0^b,c^	44.1^b,c^	40.3^b,c^	71.7
		75-79	55.6^c^	60.6^c^	27.6^b,c^	36.0^b,c^	32.7^b,c^	62.2^c^
	**Members who had ≥1 lab test in 2013 and were registered to use the patient portal during at least part of 2013**							
		65-79	79.6	82.1	60.6^b^	66.5^b^	64.1^b^	83.6
		65-69	82.4	84.8	63.7^b^	71.4^b^	70.2^b^	85.7
		70-74	80.6^c^	83.0 ^c^	61.3^c^	67.6^b,d^	64.3^b,c^	84.9
		75-79	75.7^c^	78.3 ^c^	56.4^b,c^	60.9^b,c^	57.3^b,c^	80.3^c^
**Ordered a prescription refill using the patient portal ≥1 time in 2013, %**								
	**All members who refilled ≥1 prescription in 2013**							
		65-79	35.0	38.6	16.5^b^	21.2^b^	18.5^b^	37.0
		65-69	42.1	46.5	21.2^b^	26.6^b^	22.9^b^	44.4
		70-74	36.5^c^	40.4^c^	17.5^b,c^	21.8^b,c^	18.1^b,c^	38.4
		75-79	28.1^c^	31.0^c^	11.9^b,c^	16.9^b,c^	15.5^b,c^	31.1^c^
	**Members who refilled ≥1 prescription in 2013 and were registered to use the patient portal at least part of 2013**							
		65-79	44.3	46.6	29.3^b^	33.0^b^	29.7^b^	44.8
		65-69	49.9	52.6	32.9^b^	38.0^b^	33.8^b^	50.8
		70-74	45.4^c^	47.8^c^	30.5^b^	33.3^b,d^	28.7^b,c^	45.2^d^
		75-79	38.3^c^	40.0^c^	24.3^b,c^	28.8^b,c^	27.1^b,c^	40.3^c^

^a^Cell percentages represent use among adults in that age, race/ethnic, or age-race/ethnic subgroup. The denominator for cell percentages in the “All” column includes all non-Hispanic white, black, Latino, Filipino, and Chinese members in that age group. See Multimedia Appendix 1 for cell denominators. Due to the very large denominators for all cells, comparisons with *P* values ≥.055 are not reported. See Multimedia Appendix 3 for detailed *P* values.

^b^Significantly differs (*P<*.001) from non-Hispanic whites within same age group after controlling for sex.

^c^Significantly differs (*P<*.01) from 65-69 age group within All or within same race/ethnic group after controlling for sex.

^d^Significantly differs (*P<*.01) from non-Hispanic whites within same age group after controlling for sex.

^e^Significantly differs (*P<*.05) from non-Hispanic whites in same age group after controlling for sex.

**Table 2:** Differences by age cohort and race/ethnic group in use of the health plan’s patient portal in 2013 among patients ages 65-79 who have diabetes, hypertension, and/or coronary artery disease^a^.

Use of the patient portal in 2013	Age	All	Non-Hispanic white	Black	Latino	Filipino	Chinese
**Was registered to use the patient portal by end of 2013, %**							
	65-79	76.9	81.5	55.2^b^	63.0^b^	61.9^b^	82.0
	65-69	82.2	86.8	63.5^b^	68.3^b^	67.3^b^	87.9
	70-74	78.5^b^	83.3^c^	56.2^b,c^	64.6^b,d^	62.7^b,c^	83.7^d^
	75-79	71.9^b^	76.5^c^	48.4^b,c^	57.9^b,c^	56.3^b,c^	77.0^c^
**Used the patient portal to send a secure message to a doctor, view lab test results, order a prescription refill, or make an appointment ≥1 time in 2013, %**							
	65-79	63.3	69.2	36.2^b^	44.5^b^	42.8^b^	71.7^e^
	65-69	70.3	76.7	44.1^b^	51.4^b^	50.5^b^	78.6
	70-74	65.4^b^	71.6^c^	37.2^b,c^	46.4^b,c^	43.2^b,c^	74.6^e,f^
	75-79	56.6^b^	61.9^c^	29.9^b,c^	38.2^b,c^	35.8^b,c^	64.9^b^
**Sent a secure message through the patient portal ≥1 time in 2013, %**							
	65-79	49.3	54.8	25.8^b^	32.6^b^	28.7^b^	52.8^e^
	65-69	56.0	62.2	32.5^b^	38.2^b^	35.5^b^	59.6
	70-74	51.2^b^	57.0^c^	26.6^b,c^	34.1^b,g^	29.2^b,c^	56.6
	75-79	43.0^b^	47.9^c^	20.4^b,c^	27.6^b,c^	22.4b^b,c^	45.1^c,g^

^a^Study population for this table is members who were in a health plan diabetes, hypertension, or coronary artery disease registry in 2013. Cell percentages represent use among adults in that age, race/ethnic, or age-race/ethnic subgroup. The denominator for cell percentages in the “All” column includes all non-Hispanic white, black, Latino, Filipino, and Chinese members in that age group. See Multimedia Appendix 1 for cell denominators. Due to the large denominators for all cells, comparisons with *P* values ≥.055 are not reported. See Multimedia Appendix 3 for detailed *P* values.

^b^Significantly differs (*P<*.001) from non-Hispanic white within same age group after controlling for sex.

^c^Significantly differs (*P<*.001) from 65-69 age group within All or within same race/ethnic group after controlling for sex.

^d^Significantly differs (*P<*.01) from 65-69 age group within All or within same race/ethnic group after controlling for sex.

^e^Significantly differs (*P<*.05) from non-Hispanic white within same age group after controlling for sex.

^f^Significantly differs (*P<*.05) from 65-69 age group within All or within same race/ethnic group after controlling for sex.

^g^Significantly differs (*P<*.01) from non-Hispanic white within same age group after controlling for sex.

The full respondent sample, after weighting, was predominantly non-Hispanic white (79.4%) and aged 70-74 (43.7%) (see Table 3). The age group composition of all five race/ethnic groups and the race/ethnic composition of all three age groups were nearly identical to those of the full sample. About 40% of the full sample was college educated, with an additional 33.7% having attended at least some college. Based on 2011 survey data for the same health plan population, 30.3% would be expected to be low income by community standards (household income ≤US $35,000/year), and 27.7% to have a household income >US $80,000/year. Most considered their health to be “good” or better, with only 18.1% rating their health “fair” or “poor.” Slightly over 70% had been diagnosed with a chronic cardiovascular condition (ie, in a diabetes, hypertension, or coronary artery disease registry), and 90% reported taking at least one prescription medication for a chronic condition.

Significant differences across age and race/ethnic groups were observed for educational attainment, income, and health status. Compared with 65-69 year olds, those aged 70-74 and 75-79 were significantly less likely to be college graduates and significantly more likely to be low income. Compared with non-Hispanic white seniors, black and Latino seniors were significantly less likely to be college graduates, whereas Filipino and Chinese seniors were significantly more likely to have college degrees. Nearly one-fourth (22.0%) of Latinos did not graduate from high school, compared with around 4% of the other race/ethnic groups. Black, Latino, and Filipino seniors were significantly more likely than non-Hispanic white seniors to be in the low-income group and significantly less likely to be in the higher income group, whereas the income distribution of Chinese seniors did not significantly differ from that of non-Hispanic white. Seniors aged 75-79 were significantly more likely to consider their health to be fair or poor than those in the younger groups, and black, Latino, and Filipino seniors were significantly more likely than non-Hispanic white seniors to consider themselves to have fair or poor health and to have cardiovascular conditions.

**Table 3:** Characteristics of survey respondents, after weighting, by age group and race/ethnicity^a^ .

		All, %	By age group, %			By race/ethnicity,%				
		65-79 (N=2602)	65-69 (n=841)	70-74 (n=878)	75-79 (n=883)	Non-Hispanic white (n=849)	Black (n=567)	Latino (n=653)	Filipino (n=219)	Chinese (n=314)
**Age group**										
	65-69	23.5	n/a	n/a	n/a	23.4	23.6	23.6	25.6	23.1
	70-74	43.7	n/a	n/a	n/a	43.8	43.3	42.3	45.0	42.0
	75-79	32.8	n/a	n/a	n/a	32.8	33.1	34.1	29.4	34.9
**Sex**										
	Women	54.1	53.8	53.9	54.5	53.8	56.9	54.8	57.1	48.2
	Men	45.9	46.2	46.1	45.5	46.2	43.1	45.2	42.9	51.8
**Race/ethnicity**										
	Non-Hispanic white	79.4	79.0	79.6	79.5	n/a	n/a	n/a	n/a	n/a
	Black	7.3	7.3	7.3	7.4	n/a	n/a	n/a	n/a	n/a
	Hispanic/Latino	5.4	5.4	5.2	5.6	n/a	n/a	n/a	n/a	n/a
	Filipino	5.2	5.6	5.3	4.6	n/a	n/a	n/a	n/a	n/a
	Chinese	2.7	2.7	2.6	2.9	n/a	n/a	n/a	n/a	n/a
**Educational attainment**										
	Non-high school graduate	5.0	3.2	3.1	8.9^b^	3.9	4.7	22.0^c^	4.7	4.1
	High school graduate/GED^d^	21.3	14.5	19.9	28.1	21.0	25.2	31.0	14.1	14.3
	Some college	23.8	33.4	36.1	30.8	34.2	45.0	27.8	22.7	24.6
	College graduate	39.9	48.9	40.9^e^	32.2^b^	40.9	25.0^c^	19.2^c^	58.4^c^	57.0^c^
**Household income in US $ in 2010** ^d^										
	≤25,000	17.7	11.9	18.7^b^	26.7^b^	15.8	26.1^c^	22.3^c^	29.3^c^	16.8
	25,001-35,000	12.6	10.0	13.2	16.5	11.9	16.3	17.5	17.5	8.7
	35,001-80,000	42.0	42.9	42.6	39.6	42.1	39.7	43.9	43.9	41.3
	>80,000	27.7	35.2	25.5^b^	17.2^b^	30.2	17.8^c^	16.3^c^	16.3^c^	33.3
**Self-rated health**										
	Very good or excellent	43.8	49.7	46.8	35.6^b^	48.3	21.2^c^	28.0^c^	25.9 ^c^	38.9^g^
	Good	38.1	34.8	38.2	40.2	35.9	48.8	39.9	49.7	44.8
	Fair or poor	18.1	15.5	15.0	24.2^b^	15.8	30.0^c^	32.1^c^	24.4^g^	16.3
History of diabetes, hypertension, coronary artery disease, heart failure, or stroke^h^		71.7	62.1	70.3^e^	80.6^b^	69.0	87.4^c^	77.8^c^	86.5^c^	70.5
Takes medication for ≥1 chronic condition		90.5	87.2	90.3	93.3^i^	89.9	95.4^c^	90.7	94.9^j^	86.8

^a^Cell percentages are based on weighted data for everyone in that age or race/ethnic group. Ns at the top of columns are the unweighted number of respondents in that group. *P* values ≥.055 are not reported. See Multimedia Appendix 3 for detailed *P* values.

^b^Significantly differs (*P<*.001) from 65-69 age group after controlling for race/ethnicity and sex.

^c^Significantly differs (*P<*.001) from non-Hispanic white after controlling for age group and sex.

^d^GED=General Educational Development (credential indicating that an individual has met high school level academic skills).

^e^Significantly differs (*P<*.05) from 65-69 age group after controlling for race/ethnicity and sex.

^f^Based on estimates from a 2011 health survey of the same health plan membership. A household income ≤$35,000 qualifies an individual for income-subsidized, low income housing.

^g^Significantly differs (*P<*.01) from non-Hispanic white after controlling for age group and sex.

^h^In ≥1 of the health plan’s chronic disease registries for these conditions.

^i^Significantly differs (*P<*.01) from 65-69 age group after controlling for race/ethnicity and sex.

^j^Significantly differs (*P<*.05) from non-Hispanic white after controlling for age group and sex.

### Seniors’ Access to Digital Technology

Although 81% of seniors aged 65-79 had a mobile phone, less than one-third (31.2%) had a smartphone, and less than half (47.2%) were able to send and receive text messages (see Table 4). Seniors ages 75-79 were less likely to have smartphones and text messaging capabilities than younger seniors. Among those seniors who had smartphones, over three-fourths were using apps. Over 80% (81.5%) of seniors had access to a desktop, laptop, or netbook computer. Fewer of the seniors had a tablet, and most tablet and smartphone owners (>90%) also had a desktop or laptop computer. Access to these devices declined with increasing age, and across all age groups, black, Latino, and Filipino seniors were significantly less likely than non-Hispanic white seniors to have these digital devices. Access to Internet at home also varied among the race/ethnic groups and declined with age across all of the race/ethnic groups. Of those who did not have home Internet, approximately 34.9% said that this was due to the cost.

### Ability to Use the Internet

Ability to use the Internet to get health information from websites or to communicate with others significantly differed by race/ethnicity and age (see Table 4). Approximately 80% of non-Hispanic white (83.9%) and Chinese (79.2%) seniors reported being able to use the Internet alone or with some help, as compared with 64.4% of black, 58.2% of Latino, and 53.2% of Filipino seniors. A similar spread of approximately 20 percentage points was observed between 65-69 year olds and 75-79 year olds. Slightly over 10% of these Internet users required help or someone else to go online for them. Overall, 7.9% of seniors said they had a physical problem that made it difficult for them to use a computer or the Internet, but this was more of an issue for the 75-79 age group (12.4% vs 5.6% of 65-74 year olds) and for non-Hispanic whites (11.4% of blacks, 11.8% of Latinos, and 16.3% Filipinos versus 6.7% of non-Hispanic whites). Nearly all (>95%) of seniors who use the Internet do so at home using a computer, although significantly higher percentages of Latino and Filipino seniors than non-Hispanic whites (but still under 10%) only do so using a mobile device (tablet, smartphone, or cellular phone). Black, Latino, and Filipino seniors who went online did so less frequently than non-Hispanic whites. Chinese seniors did not significantly differ from non-Hispanic whites in frequency of Internet use.

### Ability to Use Email

Whereas 80% of non-Hispanic white and Chinese seniors were able to send and receive email, only approximately 60% of black, Filipino, and Latino seniors were able to do so, even with help (see Table 4). Among those who used email, approximately 83.8% had their own email address and 16.2% used a shared email address or someone else’s email address. Over 90% (93.6%) of email users checked their email using a computer, laptop, or netbook, and 36.8% of email users at least sometimes used a mobile device (tablet, smartphone, or cellular phone) to access email. Only 6.2% of seniors solely used a mobile device, with no significant differences by age group or race/ethnicity. Seniors aged 65-74 were significantly more likely than 75-79 year olds to access email at least sometimes using a smartphone (27.5% vs 12.3%; *P<*.001), and blacks, Latinos, and Filipinos were significantly more likely than non-Hispanic whites (10.3%, 5.0%, 15.1% vs 1.8%, respectively; *P<*.01) to access email using a cellular phone at least some of the time. Approximately two-thirds (67.9%) of all email users checked their email at least once a day. Nearly one-fourth (23.5%) of black, Latino, and Filipino email users checked their email once a week or less.

### Ability to Perform Health Care–Related Tasks Using Digital Technology

Nearly two-thirds (64.2%) of seniors thought that they could use the patient portal on their own to send a secure message to their doctor or to look up a lab test result, 60.6% thought they could print out information or forms from a website, and 50.9% thought they could get to a website to get information or forms if given a URL verbally or in print (see Table 5). Whereas 65.4% thought they could complete a questionnaire on a computer by themselves, only 52.1% thought they could complete a questionnaire by interactive voice response (IVR) administration and 35.2% by using a touchscreen tablet at the clinic. These percentages increased by only 10-14 percentage points when we also included those who said they could do these tasks with some help. Across all of these tasks, seniors in the two older age groups were significantly less likely than 65-69 year olds to indicate being able to perform these tasks alone or with help. Similarly, black, Latino, and Filipino seniors were significantly less likely than non-Hispanic white seniors to say they could perform these tasks alone or with help, with many differences greater than 15 percentage points. Chinese seniors did not differ from non-Hispanic white seniors.

### Seniors’ Use of and Preference for Using the Patient Portal and Digital Technologies for Health Care–Related Tasks

Seniors were presented with five health care–related tasks that could be carried out using the patient portal and asked to indicate which of the methods listed they currently used or were willing to use and which method they most preferred to use. They were also asked how they preferred to receive information about health care benefits and health newsletters. Finally, they were asked how they would like to get health information and advice, in addition to getting this information directly from their doctor and other clinicians.

**Table 4:** Seniors’ access to digital devices, Internet, and email, by age group and race/ethnicity^a^.

			All	By age group			By race/ethnicity				
			65-79 (N=2602)	65-69 (n=841)	70-74 (n=878)	75-79 (n=883)	Non-Hispanic white (n=849)	Black (n=567)	Latino (n=653)	Filipino (n=219)	Chinese (n=314)
**Has access to a mobile phone (cellular phone or smartphone), %**			81.0	88.4	84.3	71.2^b^	82.2	82.8	72.6^c^	70.2^c^	77.0
	Has a smartphone		31.2	43.4	33.9^d^	18.7^b^	32.8	30.5	22.0^c^	19.6^c^	26.6^e^
	Able to receive text messages		47.2	61.5	51.4^d^	31.4^b^	47.4	53.6^f^	41.0^f^	45.1	54.9
	If has a mobile phone		60.5	71.2	63.1^g^	46.5^b^	60.5	59.6	68.4^h^	59.9	67.0
**Owns or has easy access to a computer, laptop, netbook, or tablet, %**			81.5	91.5	82.5^b^	73.1^b^	85.3	70.7^c^	63.0^c^	57.5^c^	82.8
	Desktop, laptop, or netbook		79.5	90.4	80.0^b^	71.2^b^	83.5	69.1^c^	61.1^c^	53.1^c^	79.8
	Tablet		25.1	34.3	27.4^g^	15.6^b^	27.1	16.0^c^	12.6^c^	20.1^f^	28.3
	Has home Internet		83.8	91.3	85.5^d^	76.2^b^	87.4	71.9^g^	68.4^h^	61.0^c^	84.8
**Able to use the Internet, %**											
	Able to use on own or with help		79.4	88.9	81.5^b^	68.7^b^	83.9	64.4^c^	58.2^c^	53.3^c^	79.2
	Uses on own		69.4	80.7	70.8^b^	59.4^b^	74.4	51.8^c^	48.0^c^	39.3^c^	69.4
	Uses with help or proxy uses		10.0	8.2	10.7	10.3	9.5	12.6	10.2	14.0	9.8
	If uses the Internet, how frequently goes online		(n=1886)	(n=707)	(n=637)	(n=542)	(n=714)	(n=390)	(n=410)	(n=125)	(n=247)
		Daily	64.2	70.0	64.3	58.8^d^	66.2	51.1^c^	53.7^c^	47.2^c^	66.0
		≤1x/wk	16.6	16.7	16.7	18.8	15.1	28.3^c^	25.8^c^	26.2^f^	14.4
**Able to use email, %**			(n=2594)	(n=839)	(n=876)	(n=879)	(n=848)	(n=565)	(n=650)	(n=217)	(n=314)
	Able to use by self or with help		79.3	86.1	81.2^g^	72.1^b^	83.4	63.2^c^	59.6^c^	58.5^c^	80.8
	Uses on own		70.0	80.2	71.9^g^	60.3^b^	74.7	52.6^c^	49.1^c^	43.0^c^	72.6
	Uses with help or proxy uses		9.3	5.9	9.3	11.8^d^	8.8	10.6	10.6	15.6^h^	8.2
	Has an email address		76.2	82.4	78.4	68.6^b^	80.5	60.0^c^	57.9^c^	51.3^c^	76.3
	Has own email address		63.8	68.5	66.3	57.2^b^	67.3	51.2^c^	48.2^c^	42.6^c^	65.7
	Shares an email address (may also have own)		11.3	13.5	11.4	9.5	13.0	6.2^c^	6.9^c^	4.9^h^	8.2^f^
	Uses someone else’s email address		1.8	1.4	1.7	2.2	1.4	3.1^f^	3.6^f^	3.7^f^	2.4
	If receives email, how frequently checks for email		(n=1866)	(n=682)	(n=630)	(n=554)	(n=699)	(n=377)	(n=420)	(n=124)	(n=246)
		Daily	67.9	70.4	68.6	64.8	70.0	49.5^c^	59.5^c^	56.4^h^	70.5
		≤1x/wk	13.8	12.4	12.2	17.4	12.3	24.6^c^	22.9^c^	22.2^f^	13.7

^a^Cell percentages based on weighted data for everyone in that age or race/ethnic group. Ns at top of columns are the unweighted number of respondents in that group except when analyses are restricted to a subset of that group. *P* values ≥.055 are not reported. See Multimedia Appendix 3 for detailed *P* values.

^b^Significantly differs (*P<*.001) from 65-69 age group after controlling for race/ethnicity and sex.

^c^Significantly differs (*P<*.001) from non-Hispanic white after controlling for age group and sex.

^d^Significantly differs (*P<*.01) from 65-69 age group after controlling for race/ethnicity and sex.

^e^Significantly differs (*P*=.053) from non-Hispanic white after controlling for age group and sex.

^f^Significantly differs (*P<*.05) from non-Hispanic white after controlling for age group and sex.

^g^Significantly differs (*P<*.05) from 65-69 age group after controlling for race/ethnicity and sex.

^h^Significantly differs (*P<*.01) from non-Hispanic white after controlling for age group and sex.

**Table 5:** Seniors’ perceptions of their ability to perform health care–related tasks involving digital technology^a^.

Task		All	By age group			By race/ethnicity					
		65–79 (N=2586)	65-69 (n=837)	70-74 (n=875)	75-79 (n=874)	Non-Hispanic white (n=847)		Black (n=562)	Latino (n=648)	Filipino (n=218)	Chinese (n=311)
**Send a message to doctor through the patient portal if had a question, %**											
	Could do by self	64.2	76.1	66.3^b^	52.9^c^	68.6		47.2^d^	44.3^d^	40.9^d^	63.3
	Could do by self or with help	79.7	88.2	81.7^b^	71.1^c^	82.9		67.2^d^	63.6^d^	66.1^d^	79.6
**Look up test result on the patient portal, %**											
	Could do by self	64.5	76.4	66.8^b^	52.8^c^	69.0		45.7^d^	43.6^d^	40.2^d^	68.2
	Could do by self or with help	78.4	87.3	80.5^b^	69.2^c^	81.9		63.8^d^	60.6^d^	61.2^d^	82.6
**Complete a short form or questionnaire on a computer, %**											
	Could do by self	65.4	76.0	68.9^b^	53.2^c^	69.4		51.2^d^	45.3^d^	45.0^d^	67.2
	Could do by self or with help	76.4	86.1	78.9^b^	66.3^c^	79.8		63.3^d^	59.3^d^	58.4^d^	79.7
**Complete a questionnaire using a touch screen tablet (such as an iPad) while sitting in a clinic waiting room, %**											
	Could do by self	35.2	51.4	38.1^c^	20.0^c^	37.6		28.1^d^	23.0^d^	21.6^d^	34.3
	Could do by self or with help	45.9	63.5	49.3^c^	28.7^c^	47.7		40.6^e^	34.6^d^	36.1^f^	47.4
**Answer questions about your health using your phone’s keypad (eg, Enter 1 if Always, 2 if Sometimes, 3 if Never), %**											
	Could do by self	52.1	62.5	55.0^g^	41.0^c^	54.6		49.4	37.8^d^	34.5^d^	50.3
	Could do by self or with help	59.3	70.9	61.3^b^	48.5^c^	61.0		57.3	46.2^d^	50.9^e^	59.5
**Go to a website to get information or forms using a URL (website address) given orally or in a letter, %**											
	Could do by self	50.9	66.9	52.1^c^	38.0^c^	55.2		37.9^d^	30.4^d^	24.3^d^	51.3
	Could do by self or with help	60.2	75.3	61.8^c^	47.4^c^	63.3		49.6^d^	42.6^d^	42.9^d^	65.2
**Print information or forms from a website, %**											
	Could do by self	60.6	72.0	63.2^b^	48.8^c^	65.3		46.2^d^	39.2^d^	30.8^d^	60.7
	Could do by self or with help	70.4	81.7	72.3^c^	59.9^c^	74.0		58.1^d^	50.8^d^	50.8^d^	74.3

^a^Cell percentages are based on weighted data for everyone in the age or race/ethnic group. *P* values ≥.055 are not reported. See Multimedia Appendix 3 for detailed *P* values. Ns at top of columns are the unweighted number of respondents in that group.

^b^Significantly differs (*P*<.01) from 65-69 age group after controlling for race/ethnicity and sex.

^c^Significantly differs (*P*<.001) from 65-69 age group after controlling for race/ethnicity and sex.

^d^Significantly differs (*P*<.001) from non-Hispanic white after controlling for age group and sex.

^e^Significantly differs (*P<*.05) from non-Hispanic white after controlling for age group and sex.

^f^Significantly differs (*P<*.01) from non-Hispanic white after controlling for age group and sex.

^g^Significantly differs (*P*<.05) from 65-69 age group after controlling for race/ethnicity and sex.

**Figure 1:**
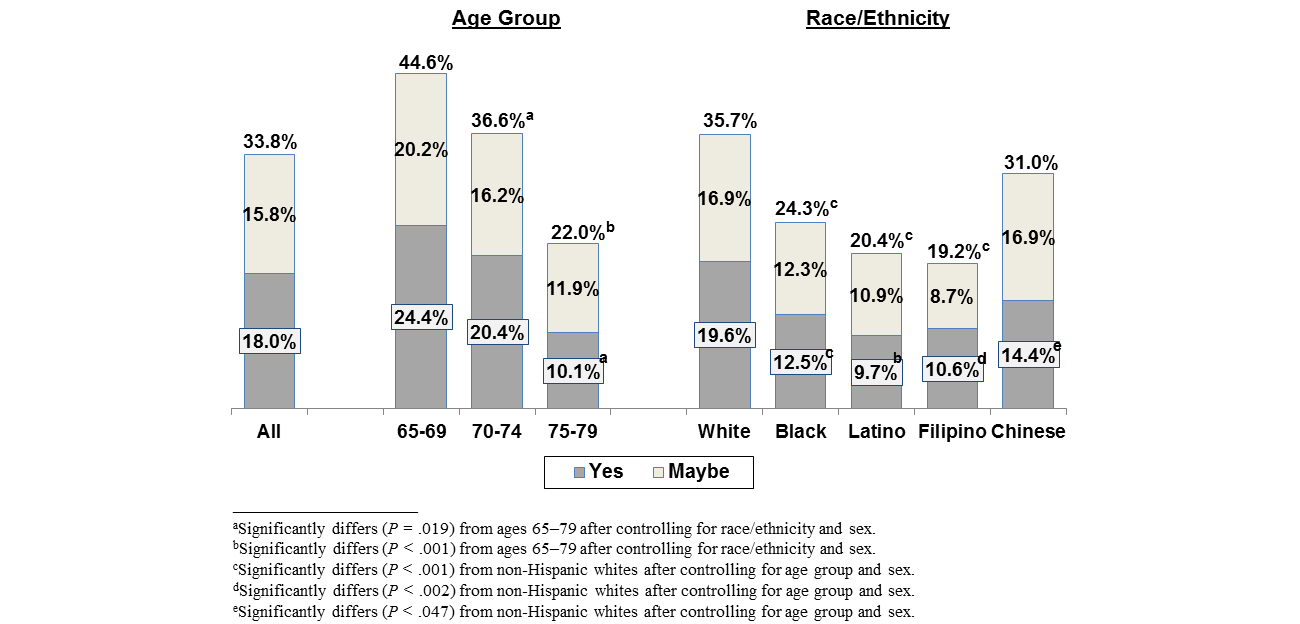
Percentages of 65-79 year olds who would be willing to have a video visit if their doctor did not think it was necessary for them to be seen in person. (A video visit enables a patient and doctor to see each other while they are talking by using a smartphone, tablet, or webcam-enabled computer).

Overall, over half (58.2%) of seniors said they send secure messages to their doctors in non-urgent situations, approximately the same percentage as communicates by phone (see Table 6). Over half (54.4%) used the patient portal to view their lab test results online, which rose to 66.7% when getting results in a secure message was included in that calculation. Significantly lower percentages of seniors used or said they would be willing to use the patient portal to order prescription refills (35.7%), to get appointment reminders via secure message (24.3%), or to complete health assessment questionnaires (49.1%). One-third of seniors said they would definitely (18.0%) or possibly (15.8%) be interested in having video visits with their doctors when the doctor did not feel it was necessary for them to be seen in person (see Figure 1).

**Table 6:** Methods used and preferred for performing tasks that could be done through the patient portal^a^_._

			All	By age group			By race/ethnicity				
			65-79	65-69	70-74	75-79	Non-Hispanic white	Black	Latino	Filipino	Chinese
**Initiate non-urgent communications with doctors**			(N=2534)	(n=822)	(n=858)	(n=854)	(n=826)	(n=555)	(n=628)	(n=215)	(n=310)
	**Send a secure message using the patient portal, %**										
		Uses this method	58.2	70.0	58.7^b^	48.8^b^	63.6	33.8^c^	36.6^c^	32.2^c^	55.6^d^
		Most prefers this method	51.8	63.9	53.8^e^	40.5^b^	57.7	25.2^c^	29.0^c^	25.0^c^	46.7^f^
	**Send a message using regular email (discouraged), %**										
		Uses this method	8.2	8.5	9.8	6.0	8.1	7.0	7.3	10.1	14.5^f^
		Most prefers this method	4.8	3.5	6.4	3.5	4.9	3.3	4.4	3.3	7.5
	**Leave phone message and get return call, %**										
		Uses this method	53.7	48.0	50.0	62.6^b^	48.8	76.9^c^	71.3^c^	75.9^c^	58.0^d^
		Most prefers this method	43.5	32.9	39.9^g^	56.0^b^	37.5	71.6^c^	67.0^c^	72.7^c^	45.8^d^
**Obtain results of lab tests**			(N=2594)	(n=838)	(n=874)	(n=882)	(n=847)	(n=566)	(n=649)	(n=219)	(n=313)
	**Look up results online using the patient portal, %**										
		Uses this method	54.4	64.9	55.4^e^	45.5^b^	58.8	31.1^c^	36.3	33.5^c^	63.6
		Most prefers this method	38.9	47.3	41.2	30.1^c^	42.9	21.4^c^	21.4^c^	15.6^c^	45.7
	**Result sent in a secure message using the patient portal, %**										
		Uses this method	32.9	35.7	35.1	27.8^g^	34.2	25.0^c^	26.1^f^	32.7	28.2
		Most prefers this method	19.1	19.6	20.4	17.2	20.4	11.1^c^	15.1^d^	19.6	10.7^c^
	**Look up results online or get in secure message using the patient portal, %**										
		Uses this method	66.6	74.4	69.3	57.5^b^	70.8	43.6^c^	48.4^c^	51.6^c^	71.4
		Most prefers this method	57.9	66.8	61.2	47.3^c^	63.1	32.3^c^	36.3^c^	35.2^c^	56.5
	**Get a letter in the mail with the result, %**										
		Uses this method	51.8	48.6	50.3	56.0^g^	47.9	71.2^c^	66.3^c^	68.3^c^	53.6
		Most prefers this method	35.6	28.7	33.1	44.0^c^	30.5	57.6^c^	54.0^c^	63.2^c^	40.4^f^
	**Have someone from call with the result, %**										
		Uses this method	18.4	16.7	17.1	21.4	17.7	27.9^c^	22.1	15.1	13.5
		Most prefers this method	7.3	5.1	6.5	9.9^g^	7.1	10.5	11.0^g^	3.9	3.8
**Order prescription refills** ^h^			(N=2258)	(n=715)	(n=764)	(n=779)	(n=731)	(n=521)	(n=561)	(n=187)	(n=258)
	**Place order online using the patient portal, %**										
		Uses this method	35.7	45.0	39.1	24.8^b^	39.7	20.0^c^	22.1^c^	12.8^c^	36.1
		Most prefers this method	33.5	42.3	37.4	21.7^b^	37.2	16.3^c^	19.8^c^	12.2^c^	34.3
	**Place order by phone, %**										
		Uses this method	63.3	58.5	59.5	71.6^b^	61.4	70.8^f^	72.3^c^	72.7^f^	59.0
		Most prefers this method	57.2	51.2	53.3	66.9^b^	55.9	61.8	64.5^f^	67.5^d^	52.8
	**Place order in person at the pharmacy, %**										
		Uses this method	20.6	18.9	19.8	23.0	17.3	37.0^c^	31.3^c^	32.6^c^	26.0^f^
		Most prefers this method	9.5	6.5	9.7	11.4^g^	7.1	22.4^c^	16.1^c^	20.9^c^	12.9^d^
**Get reminders about appointments, immunizations, etc.**			(N=2586)	(n=835)	(n=871)	(n=880)	(n=843)	(n=565)	(n=646)	(n=218)	(n=314)
	**Get a secure message using the patient portal, %**										
		Uses this method	24.3	29.2	24.4	20.6^e^	26.6	16.0^c^	16.7^c^	9.5^c^	20.6
		Most prefers this method	9.4	11.5	9.0	8.5	10.4	6.4^d^	6.0^d^	2.5^d^	8.9
	**Get a regular email, %**										
		Uses this method	44.8	54.1	48.9	32.8^b^	48.9	28.4^c^	26.2^c^	24.7^c^	44.7
		Most prefers this method	29.2	33.6	35.1	18.3^b^	33.0	12.3^c^	13.3^c^	9.3^c^	25.4^d^
	**Get an automated phone message from a computer system, %**										
		Uses this method	39.2	41.8	41.3	34.5	39.7	45.0	40.3	25.6^c^	34.0
		Most prefers this method	13.4	14.8	14.3	11.3	13.2	18.2^c^	18.7^d^	6.5^d^	11.7
	**Get a letter/postcard sent by regular mail, %**										
		Uses this method	67.8	61.9	65.4	75.1^g^	65.1	78.6^d^	75.2^c^	85.1^c^	68.6
		Most prefers this method	47.8	39.5	41.1	62.4^b^	43.2	63.5^c^	62.8^c^	80.5^c^	54.9^f^
	**Use the Kaiser Permanente preventive care app, %**										
		Uses this method	5.2	6.4	6.7	2.4^e^	5.3	5.7	4.1	5.3	3.6
		Most prefers this method	0.8	0.6	1.5	0.1	0.8	1.2	0.3	1.2	0.5
**Complete health questionnaires**			(N=2570)	(n=832)	(n=862)	(n=876)	(n=839)	(n=560)	(n=643)	(n=216)	(n=312)
	**Online questionnaire accessed via the patient portal, %**										
		Uses this method	49.1	59.1	52.0^g^	38.2^b^	54.5	28.3^c^	27.4^c^	21.1^c^	41.6^c^
		Most prefers this method	35.1	42.3	39.3	24.2^b^	39.3	19.1^c^	18.6^c^	12.8^c^	27.8^c^
	**Touchscreen tablet or computer at medical facility, %**										
		Uses this method	7.4	11.5	7.9	3.6^b^	7.8	6.7	5.1^d^	3.9^d^	6.3
		Most prefers this method	1.3	1.8	2.0	<0.1	1.5	<0.1	0.8	0.3	0.9
	**IVR questionnaire** ^i^ **, %**										
		Uses this method	12.5	11.5	13.3	12.1	12.6	17.1^d^	10.3	7.8	10.0
		Most prefers this method	2.0	2.5	1.6	2.2	1.9	3.5	2.4	2.2	1.3
	**Paper (print) questionnaire, %**										
		Uses this method	77.2	70.4	76.1	83.5^b^	75.3	85.0^c^	83.1^c^	87.4^c^	79.3
		Most prefers this method	56.6	50.1	52.4	67.0^b^	52.2	71.4^c^	71.1^c^	82.4^c^	66.6^c^
	**Interviewer administered, %**										
		Uses this method	18.5	13.2	18.8^g^	21.7^e^	18.3	24.0^d^	21.4	12.4	12.9
		Most prefers this method	5.5	3.9	5.3	7.0	5.4	6.6	8.7^d^	3.2	3.4

^a^Most preferred method restricted to people who indicated only one method or a most preferred method if >1 method was indicated. Cell percentages are based on weighted data for everyone in the age or race/ethnic group. Ns at top of columns are the unweighted number of respondents in that group. *P* values ≥.055 are not reported. See Multimedia Appendix 3 for detailed *P* values.

^b^Significantly differs (*P*<.001) from 65-69 age group after controlling for race/ethnicity and sex.

^c^Significantly differs (*P*<.001) from non-Hispanic white after controlling for age group and sex.

^d^Significantly differs (*P*<.05) from non-Hispanic white after controlling for age group and sex.

^e^Significantly differs (*P<*.01) from 65-69 age group after controlling for race/ethnicity and sex.

^f^Significantly differs (*P<*.01) from non-Hispanic white after controlling for age group and sex.

^g^Significantly differs (*P<*.05) from 65-69 age group after controlling for race/ethnicity and sex.

^h^Restricted to seniors who take medications for a chronic condition and do not rely totally on others to order their prescription refills.

^i^“By phone using the phone keypad to enter answers to questions read by a nice taped voice.”

Half of seniors were willing to get information about health care benefits (50.9%) or health newsletters (54.5%) by email (see Table 7). We found significant age and race/ethnic group differences in use of or willingness to use the patient portal or other digital technologies to conduct the five health care–related tasks (see Table 6) that paralleled some subgroup differences in perceived ability to perform these tasks (see Table 5). Older seniors were less likely to use or be willing to use the patient portal to perform some or all of these tasks than those 65-69 years old and were also significantly less willing to use video visits. For all five patient portal tasks, black, Latino, and Filipino seniors were significantly less likely than non-Hispanic whites to use or be willing to use the patient portal features instead of more traditional methods of communicating information and were also significantly less likely to be interested in video visits (see Table 6 and Figure 1). Chinese seniors were significantly less likely than non-Hispanic whites to use secure messaging and online questionnaire completion, but these differences were smaller than those of the other race/ethnic groups, and they did not differ from non-Hispanic whites on willingness to use video visits. Overall, seniors were significantly less willing to receive information about health care benefits via email or by automated calls than regular mail (50.9% and 9.4% vs 76.7%) or to get health newsletters by email versus regular mail (54.5% vs 65.9%) (see Table 7).

Of those who indicated use of any method for these health care tasks and communications, approximately 90% of seniors indicated a preferred method for communicating with doctors, ordering prescription refills, and completing health questionnaires. Around 80% had a preferred method for obtaining lab test results or receiving reminders. All indicated a health communications preference. Although seniors aged 65-69 and 70-74 were significantly more likely to prefer secure messaging with their doctor than leaving a phone message, the reverse was true for 75-79 year olds. Similarly, non-Hispanic white seniors were significantly more likely to prefer secure messaging over use of the phone, but black, Latino, and Filipino seniors were significantly more likely to prefer phone calls over secure messaging, with Chinese seniors equally split between these two options. A similar demographic pattern was observed for viewing lab test results online versus receiving them in a mailed letter. Seniors aged 65-69 were significantly more likely to order prescription refills online than by phone, but the opposite was true for the two older groups and for all race/ethnic groups. All age and race/ethnic groups significantly preferred getting reminders by regular email rather than in a secure message that required them to sign into the patient portal. With respect to completion of health questionnaires, seniors in the two older age groups and in all race/ethnic groups significantly preferred to use a print versus an online questionnaire accessed by the patient portal. Combining online and facility-based touchscreen tablet data entry (both of which enable real-time direct flow of member data into the electronic medical record) resulted in very little increase in the percentages that preferred digital questionnaires. Across all age and race/ethnic groups, seniors preferred getting health care benefit information and newsletters by regular mail than by email. Seniors in the oldest age group were significantly (*P<*.001) more likely than those in the younger groups to say they wanted to get health benefits information only by regular mail, not email (60.6% vs 40.1% and 44.9%, respectively), as were blacks (63.6%), Latinos 64.6%, and Filipinos (68.9%) compared to non-Hispanic whites (45.1%). The same differences were seen for newsletters (data not shown).

**Table 7:** Methods seniors are willing to use and would prefer for receiving newsletters and benefits information^a^.

			All	By age group			By race/ethnicity				
			65-79	65-69	70-74	75-79	Non-Hispanic white	Black	Latino	Filipino	Chinese
**Get information about benefits or other topics related to your health**			(N=2581)	(n=838)	(n=865)	(n=878)	(n=839)	(n=564)	(n=647)	(n=219)	(n=312)
	**Get an email containing all information in the body of the email, %**										
		Willing to use this method	38.6	41.9	42.5	31.1^b^	41.4	27.8^c^	27.7^c^	22.9^c^	37.5
		Most prefers this method	17.8	17.6	22.1	12.2^d^	19.8	8.6^c^	10.7^c^	8.5^c^	14.5
	**Get an email with pdf attachment, %**										
		Willing to use this method	23.3	31.9	25.9	13.7^b^	25.6	15.9^c^	12.2^c^	12.5^c^	18.1^e^
		Most prefers this method	8.9	10.6	10.5	5.6^f^	10.0	5.7^e^	3.5^c^	2.9^c^	6.7
	**Get an email with a link to a website, %**										
		Willing to use this method	22.8	34.6	22.7^b^	14.6^b^	25.1	13.8^c^	13.1^c^	13.3^c^	18.1^g^
		Most prefers this method	7.7	12.9	6.9	5.1	8.8	2.9^c^	4.1^g^	1.2^c^	5.7
	**Get the information by one or more of the above types of emails, %**										
		Willing to use this method	50.9	59.5	55.0	39.3^b^	54.8	36.0^c^	34.0^c^	30.7^c^	47.9^h^
		Most prefers this method	34.3	41.1	39.5	22.8^b^	38.6	17.3^c^	18.4^c^	12.7^c^	26.9^g^
	**Get print information by regular mail, %**										
		Willing to use this method	76.6	74.0	73.3	82.7^f^	74.4	87.1^c^	84.0^c^	87.2^c^	76.9
		Most prefers this method	60.7	52.6	56.4	72.1^b^	57.3	73.3^c^	75.3^c^	78.8^c^	64.1^e^
	**Get an automated phone message** ^i^ **, %**										
		Willing to use this method	9.4	9.6	8.8	9.9	9.1	13.8^g^	13.0^g^	4.4^g^	6.4
		Most prefers this method	0.6	0.9	0.3	0.7	0.4	1.8^g^	2.5^e^	0.4	<0.1
**Get health newsletters** ^i^			(N=2377)	(n=769)	(n=790)	(n=818)	(n=815)	(n=480)	(n=594)	(n=187)	(n=301)
	**Get an email containing the newsletter in the body of the email, %**										
		Willing to use this method	39.2	44.0	44.5	28.5^b^	42.8	25.9^c^	24.0^c^	21.2^c^	33.4^g^
		Most prefers this method	21.6	22.6	25.5	16.0^d^	23.9	11.4^c^	13.3^c^	11.7^c^	17.3^g^
	**Get an email with a pdf attachment, %**										
		Willing to use this method	23.8	31.8	25.3^d^	16.0^b^	26.4	16.0^c^	12.4^c^	11.2^c^	15.5^c^
		Most prefers this method	10.4	11.9	12.1	7.0^d^	11.8	6.7^e^	5.0^c^	1.4^c^	7.1^g^
	**Get an email with a link to a website, %**										
		Willing to use this method	24.0	32.2	25.2^d^	16.4^b^	26.3	14.9^c^	13.7^c^	14.2^c^	21.9
		Most prefers this method	9.5	13.9	10.4	5.3^b^	10.5	5.5^e^	5.1^g^	4.8^g^	10.5
	**Get the newsletter in ≥1 of email types, %**										
		Willing to use this method	54.5	62.5	59.3	42.2^b^	59.3	37.2^c^	34.4^c^	30.0^c^	48.2^e^
		Most prefers this method	38.4	44.0	44.2	26.6^b^	42.7	21.2^c^	22.1^c^	16.5^c^	32.0^e^
	**Get a print newsletter by regular mail, %**										
		Willing to use this method	65.9	62.0	60.5	75.8^b^	62.7	80.3^c^	77.8^c^	80.8^c^	68.1
		Most prefers this method	58.7	51.9	52.3	71.7^b^	54.1	76.4^c^	76.6^c^	82.2^c^	65.1^e^

^a^Most preferred method restricted to people who indicated only one method or a most preferred method if >1 method was indicated. Cell percentages are based on weighted data for everyone in the age or race/ethnic group. Ns at top of columns are the unweighted number of respondents in that group. *P* values ≥.055 are not reported. See Multimedia Appendix 3 for detailed *P* values.

^b^Significantly differs (*P*<.001) from 65-69 age group after controlling for race/ethnicity and sex.

^c^Significantly differs (*P*<.001) from non-Hispanic white after controlling for age group and sex.

^d^Significantly differs (*P*<.05) from 65-69 age group after controlling for race/ethnicity and sex.

^e^Significantly differs (*P*<.01) from non-Hispanic white after controlling for age group and sex.

^f^Significantly differs (*P*<.01) from 65-69 age group after controlling for race/ethnicity and sex.

^g^Significantly differs (*P*<.05) from non-Hispanic white after controlling for age group and sex.

^h^Differs (*P*=.050) from non-Hispanic white after controlling for age group and sex.

^i^Restricted to people who completed the longer form of the questionnaire.

Willingness to go online to perform health-related tasks was significantly higher among those who could use the Internet on their own or with some help than in the overall senior population, with the same patterns of significant age group and race/ethnic differences as seen for other measures (see Table 8). However, even among those seniors who were able to use the Internet to get health information from websites or to communicate, many were not willing to perform health care–related tasks online. When we linked the subset of survey respondents who indicated being able to use the Internet (alone or with help) to evidence that they or a proxy had used at least one patient portal secure feature (sending a secure message, viewing lab test results online, ordering a prescription refill, or making an appointment) in 2013, we did not find significant age group differences (84.0%, 84.0%, and 80.3%, for the 65-69, 70-74, and 75-79 age groups, respectively) but did find significantly (*P*<.001) lower usage among black (60.0%), Latino (74.0%), and Filipino (72.3%) seniors than non-Hispanic white (85.3%) or Chinese seniors (85.7%), which remained even after adjusting for age and educational attainment.

**Table 8:** Willingness to perform health care–related tasks online^a^.

Health care–related tasks		All	By age group			By race/ethnicity				
		65-79	65-69	70-74	75-79	Non-Hispanic white	Black	Latino	Filipino	Chinese
**Currently communicates at least sometimes with doctor using secure messaging when not urgent, %**										
	All	58.2	70.0	58.7^b^	48.8^b^	63.6	33.8^c^	36.6^c^	32.2^c^	55.6^d^
	Those who can use the Internet	71.8	77.9	70.3^b^	68.5^b^	74.6	51.0^c^	58.8^c^	58.1^c^	68.9
**Currently views lab test results online at least sometimes, %**										
	All	54.4	64.9	55.4^e^	45.5^b^	58.8	31.1^c^	36.3^c^	33.5^c^	63.6
	Those who can use the Internet	67.1	72.9	66.3^b^	63.2	69.0	47.4^c^	57.9^c^	60.0^c^	75.3
**Currently orders prescription refills online at least sometimes** ^f^ **, %**										
	All	35.7	45.0	39.1	24.8^b^	39.7	20.0^c^	22.1^c^	12.8^c^	36.1
	Those who can use the Internet	44.4	50.2	47.8	34.2^b^	46.8	29.9^c^	36.3^c^	22.8^c^	43.4
**Willing to complete health questionnaires online, %**										
	All	49.1	59.1	52.0^g^	38.2^b^	54.5	28.3^c^	27.4^c^	21.1^c^	41.6^c^
	Those who can use the Internet	61.5	66.6	63.5	53.7^b^	64.7	43.7^c^	45.3^c^	39.4^c^	51.5^c^
**Willing to complete health questionnaires in the clinic using a tablet or touchscreen computer, %**										
	All	7.4	11.5	7.9	3.6^b^	7.8	6.7	5.1^d^	3.9^d^	6.3
	Those who can use the Internet	9.1	13.0	9.4	5.1^b^	9.3	9.5	7.8	5.6	7.9
**Willing to read health information online at health plan or other website, %**										
	All	35.4	42.4	38.4	26.4^b^	39.1	22.4^c^	20.5^c^	15.9^c^	28.3^g^
	Those who can use the Internet	44.3	47.7	46.5	37.6^b^	46.4	34.0^c^	33.6^c^	29.8^c^	34.9^c^
**Willing to watch health videos online at health plan website or another website like YouTube, %**										
	All	24.5	27.4	27.9	18.0^e^	26.7	17.5^c^	15.2^c^	11.6^c^	24.3
	Those who can use the Internet	30.5	30.7	33.4	25.7	31.4	26.8	24.9^c^	20.2^c^	30.3
**Willing to consider (“yes” or “maybe”) a video visit with doctor instead of an office visit, %**										
	All	33.8	44.6	36.6^e^	22.0^b^	36.5	24.8^g^	20.6^g^	19.3^g^	31.6
	Those who can use the Internet	41.7	49.5	44.8	29.5^b^	42.8	37.7	32.8^g^	34.3^d^	36.0
**Willing to get health care–related information by email (in body of email, pdf attachment, or link), %**										
	All	50.9	59.5	55.0	39.3^b^	54.8	36.0^c^	34.0^c^	30.7^c^	47.9
	Those who can use the Internet	63.5	68.1	67.8	53.5^b^	65.1	56.3^c^	55.5^c^	52.9^c^	58.1

^a^Ability to use the Internet was assigned based on a “Yes” answer to the question “Can you use the Internet to get information from websites or to communicate with others?” Most senior Internet users were able go online on their own, but some indicated needing help or someone to go online for them. Ability to use email was assigned using the same type of question and responses. Cell percentages are based on weighted data for everyone in that age or race/ethnic group. Because percentages are based on responses to different questions, unweighted cell Ns vary. Most cell Ns can be ascertained from earlier tables, and they are also provided in Multimedia Appendix 4. *P* values ≥.055 are not reported. See Multimedia Appendix 3 for detailed *P* values.

^b^Significantly differs (*P*<.001) from 65-69 age group after controlling for race/ethnicity and sex.

^c^Significantly differs (*P*<.001) from non-Hispanic white after controlling for age group and sex.

^d^Significantly differs (*P*<.05) from non-Hispanic white after controlling for age group and sex.

^e^Significantly differs (*P*<.01) from 65-69 age group after controlling for age group and sex.

^f^Restricted to seniors who take medications for a chronic condition and do not rely totally on others to order their prescription refills.

^g^Significantly differs (*P*<.01) from non-Hispanic white after controlling for age group and sex.

### Characteristics of Seniors Who Are Not Using the Patient Portal

About 40.53% (93,667/231,080) of the seniors in the full study population did not use (or have a proxy use on their behalf) any of four patient portal features (secure message to a doctor, viewing lab test results online, ordering a prescription refill, or making a primary care or vision care appointment) in 2013. Of these non-portal users, 80.20% (75,120/93,667) were 70-79 years old (38.89%, 36,426/93,667, aged 75-79), and over half (56.49%, 52,911/93,667) had not registered for a patient portal account.

To learn more about nonusers of the patient portal, we linked survey respondents with their 2013 patient portal utilization data. We found that among those who had not used any of the four portal features in 2013, 56.6% did not use the Internet even with help, 12.8% used it but needed help or someone else to use it for them, and 30.6% were able to use it on their own. Latino and Filipino nonusers of portal features were significantly more likely than non-Hispanic white nonusers (70.1%, 73.5% vs 52.5%, respectively, *P*<.001) to be unable to use the Internet even with help, whereas black (56.1%) and Chinese (56.0%) nonusers of portal features did not significantly different from non-Hispanic whites. Seniors in the 75-79 age group who had not used the patient portal were significantly more likely than non-portal users aged 65-69 (65.2% vs 42.5%, *P*<.001) to be non-Internet users, with 54.1% of 70-74 year old non-portal users lacking ability to use the Internet. Over 40% (44.0%) of non-portal users had easy access to a device (desktop or laptop computer, netbook, tablet, or smartphone) that could be used to access the Internet. Yet, only one-third (34.3%) had gotten health information from a website in the previous 12 months, and only 24.7% thought that they could get to a website given a URL in printed material or an oral message even with help.

**Figure 2:**
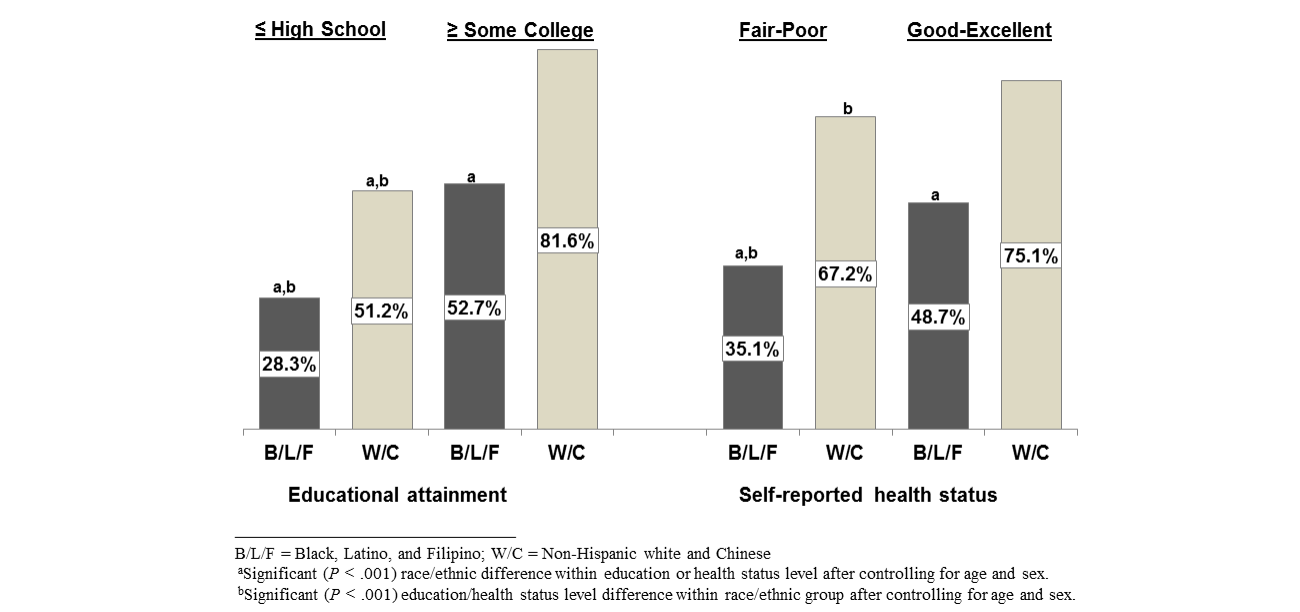
Use of patient portal features in 2013 is significantly lower among seniors with ≤ high school education and in fair-poor health, and lower among Black, Latino, and Filipino seniors in these vulnerable groups.

Seniors with a high school education or less were significantly less likely to have used any of the four patient portal features than those with at least some college or with a 4-year college degree (46.0% vs 71.6% and 81.6%, respectively, *P*<.001 for both comparisons). Seniors who considered their health to be fair or poor were significantly less likely than those with good to excellent health to have used these portal features (58.0% vs 71.0%, *P*<.001). Blacks, Latinos, and Filipinos had very similar rates of any portal use within the education and health status categories, as did non-Hispanic white and Chinese seniors, so we collapsed the five race/ethnic groups into two for comparison of education and health status by race/ethnicity. Black, Latino, and Filipino seniors were significantly less likely to use the portal than non-Hispanic white and Chinese seniors with and without formal education beyond high school (Figure 2). Black, Latino, and Filipino seniors who considered their health to be fair or poor were also significantly less likely than non-Hispanic white and Chinese seniors with similar health status to use patient portal features. They were also significantly less likely than black, Latino, and Filipino seniors with good to excellent health to use patient portal features. Use of any of the patient portal features did not significantly differ by health status for non-Hispanic white and Chinese seniors.

Of the 26% (288/843, unweighted) of non-portal users who expressed interest in learning how to use patient portal features, 50.4% were currently unable to use the Internet by themselves, and 25.3% did not have easy access to a digital device to go online.

### Impact of the Shift to Greater Use of Patient Portals and Online Communication

Seniors were asked whether, in their opinion, the health plan’s shift toward using its website has made it easier or harder for them to perform five health care–related tasks (getting information about health plan benefits and costs, communicating with their doctor, getting lab test results, getting information about health conditions and treatments, and getting health education) and overall managing their health care. Results are shown in Table 9. Most seniors (47-75%) felt that these tasks had become easier, but a sizable minority (11-17%) thought that it had become harder. Seniors aged 75-79 were significantly more likely than 65-69 year olds, and black, Latino, and Filipino seniors were significantly more likely than non-Hispanic white seniors to think that it had gotten harder for them to do all of these tasks. Chinese seniors did not significantly differ from non-Hispanic whites. When we adjusted for ability to use the Internet by oneself, the race/ethnic differences became statistically insignificant, but the age group difference remained significant.

**Table 9:** Seniors’ opinions on the effect of technology on ease of health care communication and education^a^_._

Health care–related tasks		All	By age group		By race/ethnicity				
		65-79	65-74	75-79	Non-Hispanic white	Black	Latino	Filipino	Chinese
**Get information about your health plan benefits and costs, %**									
	Easier	46.8	50.2	38.9^b^	46.1	48.0	47.2	53.7	50.5
	Harder	16.8	13.8	23.7^c^	15.0	20.9^d^	23.4^e^	29.8^f^	21.1^f^
**Communicate with your doctor, %**									
	Easier	73.3	76.5	65.9^g^	76.1	58.2^f^	63.2^f^	61.4^f^	69.3
	Harder	11.8	9.6	17.0^g^	10.1	16.7^d^	18.7^f^	24.4^f^	14.6
**Ability to get lab test results, %**									
	Easier	74.8	77.5	68.6^b^	77.2	60.0^f^	65.6^f^	64.9^d^	74.8
	Harder	11.1	9.1	15.7^b^	9.5	16.6^e^	17.1^f^	21.0^f^	14.2
**Get information you want about health conditions and treatments, %**									
	Easier	59.5	62.6	52.4^b^	60.3	53.3^h^	57.3	56.4	62.0
	Harder	12.9	10.6	18.2^g^	11.0	18.0^d^	18.4^d^	26.8^f^	18.3^h^
**Get health education to help you improve your health or reduce risks, %**									
	Easier	57.6	61.5	48.2^g^	58.3	52.5	54.3	56.6	57.8
	Harder	12.4	10.1	17.8^b^	10.5	16.8^d^	20.0^f^	25.6^f^	17.1^h^
**Manage your health care, %**									
	Easier	61.7	66.2	50.8^g^	63.2	53.5^d^	56.5^i^	54.4^h^	61.7
	Harder	12.1	10.0	17.3^b^	10.5	15.5^c^	19.0^f^	23.9^f^	15.4

^a^Seniors were asked whether the health plan’s shift toward using its website and patient portal has made it easier or harder for them to obtain information and communicate with their doctors. Analyses were restricted to people who expressed an opinion (including that there had been no change) about their ability to perform this task. Cell percentages are based on weighted data for everyone in the age or race/ethnic group. Because people did not indicate opinions about all tasks, unweighted cell Ns vary; they are provided in Multimedia Appendix 4. *P* values ≥.055 are not reported. See Multimedia Appendix 3 for detailed *P* values.

^b^Significantly differs (*P*<.01) from 65-74 age group after controlling for race/ethnicity and sex.

^c^Significantly differs (*P*<.05) from 65-74 age group after controlling for race/ethnicity and sex.

^d^Significantly differs (*P*<.01) from non-Hispanic white after controlling for age group and sex.

^e^Significantly differs (*P*=.050) from non-Hispanic white after controlling for age group and sex.

^f^Significantly differs (*P*<.001) from non-Hispanic white after controlling for age group and sex.

^g^Significantly differs (*P*<.001) from 65-74 age group after controlling for race/ethnicity and sex.

^h^Significantly differs (*P*<.05) from non-Hispanic white after controlling for age group and sex.

^i^Significantly differs (*P*=.053) from non-Hispanic white after controlling for age group and sex.

## Discussion

### Principal Findings

Our study found that in 2013, nearly 80% of adults aged 65-79 in a large integrated health care delivery system in Northern California were able to use the Internet and email, had easy access to computers, mobile phones, and home Internet, and were using the health plan’s patient portal. The percentages of Internet users in our three senior age groups were not much higher than those found in the 2012 Pew Internet Project national survey of seniors [22]. Significantly, both our patient portal utilization and survey results affirm that the shift to eHealth has the potential to limit access to two-way exchange of health information for segments of the older adult population who are already more vulnerable to chronic health problems, health care access barriers, and likely poorer outcomes.

Specifically, among seniors who had been health plan members for over 2 years, we documented age and race/ethnic disparities among KPNC members aged 65-79 in registration for and actual use of the health plan’s patient portal secure features during the 2013 calendar year, even though all members were being actively encouraged at multiple touch points (eg, clinicians, receptionists, and electronic and mail media communications) to sign up for and use the patient portal. Using survey data linked to patient portal registration and utilization, we showed that race/ethnic disparities in use of the patient portal were present even among seniors who have the ability to use the Internet. We also showed that seniors with a high school education or less and those who were in fair or poor health were less likely to have used the patient portal than better educated and healthier seniors, respectively, and that within the more vulnerable groups, blacks, Latinos, and Filipinos were less likely to be portal users than non-Hispanic white and Chinese seniors. Previous studies have found similar race/ethnic and age-related disparities in the use of health plan patient portal features by seniors [24,32,33], but our study uses more current patient portal utilization data and focuses on differences within the senior age group.

Our research found significant differences between ethnic Filipino and Chinese seniors in their use of the health plan patient portal and their ability to use and preferences for using the Internet for health-related purposes, with ethnic Chinese seniors in most cases looking similar to non-Hispanic white, and ethnic Filipinos looking more similar to blacks and Latinos. These two ethnic groups are usually combined along with other Asian ethnicities into a broad “Asian” race/ethnic group. Our results suggest that doing so may be misleading for purposes of planning roll-outs of services and dissemination of health information, resulting in inequities across multiple Asian subgroups.

More importantly, we demonstrated that descriptive statistics about Internet access and preferences for digital engagement that are based on seniors as a group and not broken out by age cohorts and race/ethnicity within age cohorts can provide a deceptively optimistic picture of seniors’ readiness to engage with patient portals and Web-based information than is the reality for certain segments of the senior population, specifically those who are older, non-white, less educated, and lower income. Evaluation of portal use among the population segments with lower ability/desire to use Internet-based communication will require that researchers pay attention to population sampling and post-stratification weighting of respondent data in the absence of data on the full population, such as we employed for our comparisons of account registration and utilization of patient portal features. In conducting our research, we also found that black, Latino, and Filipino seniors, especially those who had not signed up to use the health plan’s patient portal, were significantly less likely to respond to our survey than non-Hispanic white and Chinese seniors, who were significantly more likely to be digitally connected and using the patient portal. This suggests that studies concerned with profiling eHealth engagement in multi-ethnic senior populations or specifically studying racial or ethnic differences need to employ stratified random samples that oversample these race/ethnic groups, not only because individually they tend to make up a smaller percentage of the total senior population, but also because seniors in these race/ethnic groups are much less likely to respond to a research survey. This also extends to evaluating Internet and eHealth use and preferences in populations that include other vulnerable subgroups, such as people with low income and low educational attainment.

Ability to use the Internet and having an email address are basic requirements for registering for a patient portal account that enables a member to access secure portal features, complete online health plan questionnaires that feed responses directly into the electronic medical record, and have secure email interactions with health care providers and other health plan staff. As more information and health care–related transactions become available through patient portals and health plan websites, and assumptions are made by health plan medical staff and workflow planners that most adult members will migrate to Web-based interactions, seniors who cannot or do not want to use their health plan’s patient portal and website may find it harder to interact and access information and services. In our survey, less than half of seniors who had not used any of the four major patient portal features during the year prior to the survey were able to use the Internet or email even with someone’s help and one fourth did not have access to a device that could be used to go online. In addition to disparities in Internet and email access, we found that the majority of black, Latino, and Filipino seniors and close to half of 75-79 year olds did not think they would be able to perform many of the most common health care–related tasks that could be done using the patient portal and health plan website. Further, we found that these race/ethnic and age group differences in perceived ability to use and preference for using the patient portal and website for these tasks persisted even among those seniors who were Internet users. This suggests that successful efforts to reduce race/ethnic- and age-related disparities among seniors in use of patient portals and other eHealth modalities and thus reduce the risk of exacerbating disparities in health and health care access will require more than increasing access to the Internet through community-based WiFi or increasing efforts to promote patient portal registration and use.

**Figure 3:**
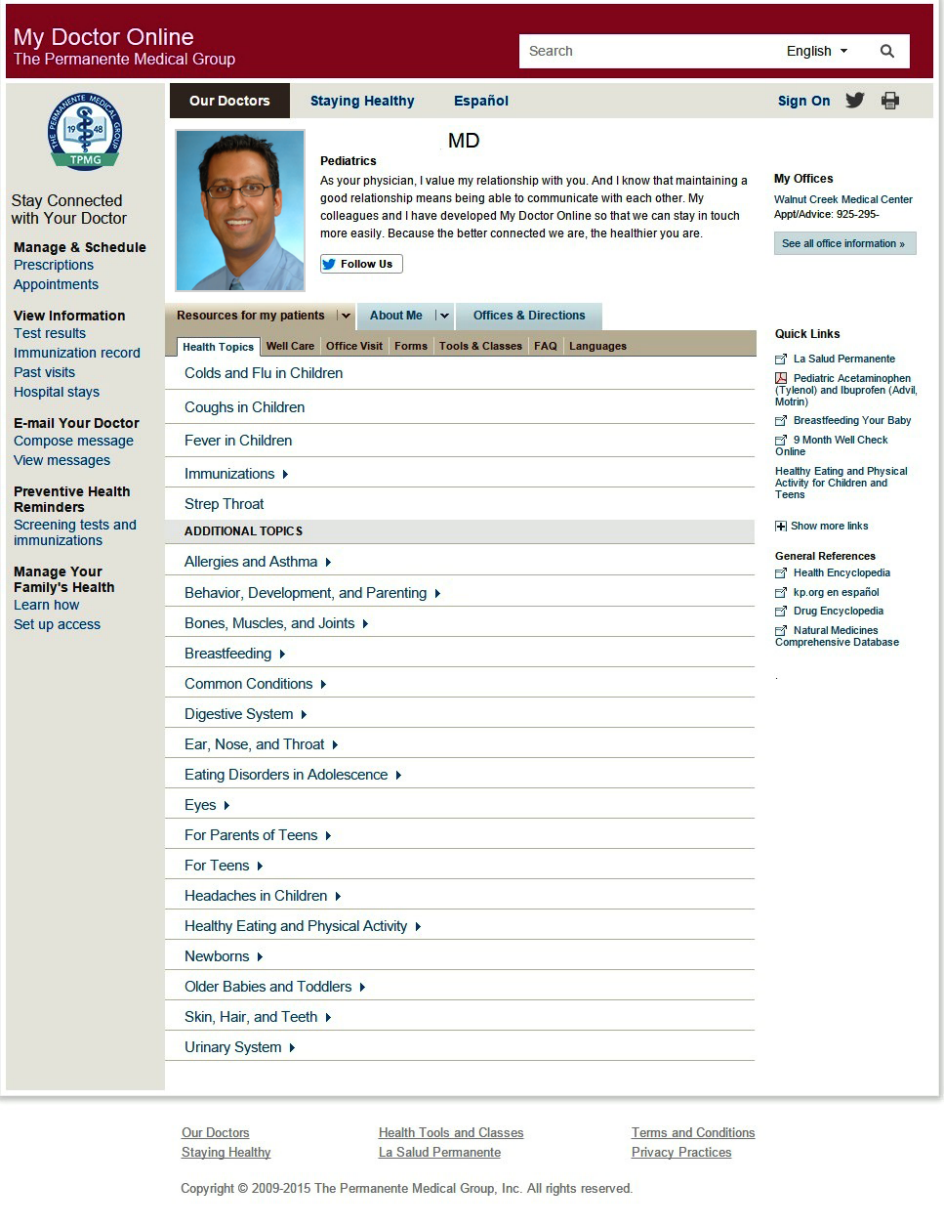
Screenshot of a Doctor Home Page developed by The Permanente Medical Group to make it easier for health plan members to use the health plan's website and patient portal.

Although some seniors who were not using patient portal features or the health plan website say they would be willing to do so if required by the health plan, they also indicated that they needed to have a person (not a Web-based video or guide) provide instruction and support for using these Web-based tools. A 2013 survey from the Pew Research Center found that 66% of non-Internet-using seniors would require help from another person to go online [21]. The same survey also found that among non-Internet-using seniors, only 13% thought they would be knowledgeable enough to go online by themselves, and only 5% of seniors said they were likely to start using the Internet or email in the future [21]. While older adults have been found to have less trust in the Internet as a source of health information [44], trust is likely less of an issue with using a health plan website. A major barrier is that most seniors, and especially those aged ≥75, will be what Prensky has termed “digital immigrants” to Web-based health care interactions, having had limited, if any, experience using computers and the Internet during their school and work years [45]. In contrast, most websites and patient portals are created by “digital natives” for use by a majority “digital native” adult population—not for older and low eHealth-literate adults. Many age-related cognitive, physical, and psychomotor factors specific to older adults can make it difficult for them to use digital technologies in general and to feel that the effort required to learn to use a complex, hierarchically designed health plan website will outweigh the benefits of using it [22,46]. Morrow and Chin [46] make a number of evidence-based recommendations for how to design patient portals and websites to make them easier for older adults to use, including organizing information and tasks in a way that is consistent with older adults’ expectations, simplifying website navigation with shallow menus and quick links, making it simple to perform common portal tasks that help them manage their health and communicate with their providers, and using fonts and formats that are easy for older eyes to read. Many of the recommendations apply more generally to making the process of interacting with websites and patient portals easier for all patients with low eHealth literacy. Because older seniors are more likely to use a computer to go online than a smartphone or tablet [47], apps designed to make it easier to use patient portal functions with a smartphone or tablet will not work as well as a landing page with easily identifiable links to frequently used patient portal features and patient education resources for reducing navigation difficulties. A good example is the Permanente Medical Group’s My Doctor Online Physician Home Page, which was created to make it easier for Kaiser Permanente Northern California Region health plan members to access patient portal functions and patient education resources on the health plan’s complex website. (see Figure 3).

Advances in Web design, digital technologies, and greater availability of free Internet access outside the home are increasingly making it easier for older adults with poor eyesight, physical disabilities, and little computer and Internet experience to go online for health. Also, websites continue to improve based on user feedback. However, if seniors are not aware of these advances or do not receive the training and support they need, they may not attempt to use these tools, especially if they had negative experiences in the past. Watkins and Xie recommend tailoring eHealth literacy interventions to take into account known learning styles of different senior demographic subgroups as well as the starting level of experience in using the Internet rather than using a one-size-fits-all approach [48]. Evaluations of in-person eHealth training programs for seniors have shown positive changes in attitudes, skills, and use of Internet-based resources for obtaining health information [49,50]. Our study results suggest that training and ongoing support for those who want to use patient portals, websites, and other eHealth technologies will be easier for the majority of seniors to access if made available in the form of hardcopy (paper) handbooks, in-person workshops or tutorials, and toll-free call-in support, not just Web-based resources.

Some seniors in our survey who do not use the Internet expressed concern that they will miss important information that is readily available only on the website or via emails and that they will lose the ability to handle their health care-related tasks without having a relative or informal caregiver act as their intermediary. Some also indicated a fear that as Web-based health care interactions become more the norm, they are going to lose the in-person and phone-based interactions with their doctors and other staff that they feel are important to nurturing their relationships with their health care providers. This is consistent with “digital immigrants” having different expectations and preferences for how they want to interact with their health care providers and the health care system that may not align with what is not only acceptable to but desired by the “digital native” majorities of adult health plan members and health care providers. Our survey results suggest that the eHealth digital divide is already causing significant percentages of black, Latino, Filipino, and older seniors to feel that a shift toward website-based health communications on the part of their health plan has made it harder for them to access information and communicate with their doctors. This is especially concerning because as our study and other research [51] suggests, compared with non-Hispanic whites and Asians, higher proportions of black and Latino seniors have chronic health problems, poorer health, and greater disability [52] and also have low levels of health literacy [53].

Health care organizations and government programs will need to take into account differences in technology access and communication/transaction preferences when designing and implementing health and health care-related communication strategies for culturally and economically diverse adult populations with a wide age range. Although the Internet and other digital technologies offer convenience and access to a greater amount of health-related information, self-care resources, and services than people have had in the past and will play a major role in Health 2.0 [54], health care organizations will need to continue to make similar resources available in more low-tech modalities (print information, DVDs, phone, regular mail) for those who are unable or do not want to access these resources from websites and email.

Health care organizations should expect that some segments of the senior population will prefer not to become “digital immigrants” and want to continue to communicate about health-related matters and engage in other types of health care-related transactions in person, by phone, and using hardcopy print rather than electronic materials. Morrow and Chin suggest that in this regard, it is very important for health care providers to send a clear message to senior patients and their family members that patient portals, secure email, Web-based patient education resources, apps, and other eHealth modalities are meant to supplement, not supplant the modes of personal patient-provider relationship that many seniors value [46]. For example, leaving a voicemail message for a doctor should be as easy as emailing that doctor through the patient portal. Additionally, health care providers need to ensure that limited digitally proficient seniors do not feel pressure to arrange for a family member or friend act as their digital interpreter if they cannot or do not want to engage with their health care providers and health plan using a patient portal. Encouraging use of a digital interpreter not only risks undermining the sense of autonomy of otherwise cognitively competent seniors to manage their own care [55] but also raises many of the same communication-related concerns that have led to recommendations against using family members and friends as medical interpreters for patients with language barriers [56].

### Strengths

Our study had several strengths. First, the patient portal component of this study was done using an extremely large and diverse cohort of Medicare-age health plan members. The health plan itself represents an integrated health care delivery system with a highly developed website and patient portal. This enabled us to compare age-group and race/ethnic differences in patient portal use in a population where all members had received extensive encouragement to sign up for the patient portal over at least 2.5 years and had access to the same health care system and patient portal.

Second, by linking patients’ electronic medical record data with use of patient portal features, we were able to restrict comparisons of use of the patient portal for online viewing of laboratory test results and online ordering of prescription refills to seniors who would have had cause to perform these tasks—something that has not been done in previous studies with this or other health plan populations.

Third, because of the size and diversity of the cohort, we were able to document significant age-group disparities and race/ethnic disparities within age groups in this Medicare-age population using directly observed percentages, not just odds ratios from logistic regression models. We were similarly able to document that these disparities persisted even among those who were registered to use the patient portal and among those with chronic health conditions who might be expected to have greater need for engaging with the health care system.

Fourth, our survey sample enabled us to compare access to and ability to use digital devices and the Internet, as well as experience with and preferences for performing health care–related tasks using digital technology across age cohorts and race/ethnic groups in a way that has not been done for a population of adults aged 65 and older. We were also able to link our survey data to patient portal registration and utilization data. This resulted in our discovery of a differential survey response rate by patient portal account creation status (our proxy for Internet access in the portal study component), which we subsequently incorporated into the survey weighting factor. This also made it possible for us to examine social determinants of use of a patient portal using real utilization data and self-reported social determinant variables such as education and Internet access.

Fifth, we were able to differentiate between patients’ use of or willingness to use digital technology for health care–related tasks and their preferences for using these technologies in general.

### Limitations

One limitation of the patient portal component of this study is that we did not have information for the full study population about overall Internet access practices and other factors such as education and income to determine whether disparities were due to these types of social determinants or to patient preferences. As a proxy for the propensity to use the Internet, we compared use of patient portal features among seniors who had created a patient portal account in 2013; this is similar to what has been used by other studies [13,18]. As described above, we also used survey data for a subset of this study population to examine whether disparities in patient portal utilization persisted within different levels of education, health status, and ability to use the Internet.

Although we were able to examine whether differences in use of secure features during the observation year persisted among those who had signed up to use the patient portal by the end of the year, we were not able to determine whether the need to obtain laboratory test results or refill prescriptions occurred before or after members created their patient portal account. We assumed that all members would have had the opportunity to use the portal to perform these tasks had they desired because everyone in this study population had, by design, been a member for at least 18 months before the study period and could have immediately activated their patient portal account when they created it.

The response rate to the survey was lower than we desired, especially among black, Latino, and Filipino seniors, which limited our ability to study race/ethnic differences within age groups. The small numbers in these groups may also limit generalizability. The numbers of Filipino and Chinese seniors included in the survey were also smaller than we would have liked because these ethnic groups had originally been selected only for pilot study purposes. Had the analysis of patient portal use in the full study sample been completed prior to the survey, we likely would have included comparable numbers of Filipinos and Chinese in the sample to increase the precision of our statistics and to have more statistical power to test for differences in access and preference between these two Asian ethnic groups.

We did not include a question about personal or family income in the survey because a large percentage of seniors had left the income question blank in previous health plan surveys or been disconcerted about being asked. We did ask whether cost was a factor in not having Internet at home and used self-reported education as a measure of socioeconomic status. We also used income data from a 2011 KPNC Member Health Survey to characterize income differences among the age and race/ethnic groups in this health plan, but we were not able to shed light on the joint effect of education and income on access to and preference for using eHealth technology.

Finally, no validated measures of health literacy were included in our survey, so we were unable to study the extent to which health literacy mediates differences in seniors’ access to and preferences for using health information technology as part of their health care.

### Conclusions

Our study documents digital disparities by age, race/ethnicity, and educational attainment within the senior age group with regard to access to digital devices, ability to use the Internet and email, and preferences for going online or using traditional telephones to interact with health care providers and the health care system in the United States. Our results suggest that the same subgroups of vulnerable seniors that have previously been shown to have difficulties with health care access may also be hampered by the eHealth digital divide from obtaining timely health information and advice, using digital monitoring devices as part of chronic disease self-management, and taking advantage of cost-saving Internet-based care options such as online purchase of prescription medications and medical equipment and having video visits with doctors and patient educators. Because well-known disparities in health status and health care access and use are being extended into the eHealth arena, we do not expect digital technologies to reduce socioeconomic gradients automatically.

In order to ensure that eHealth disparities do not increase health status and health care access disparities between more privileged and less privileged groups, eHealth initiatives should embed tracking systems and measures of disparities in their access and use. Health care delivery systems, government agencies, and other organizations that serve multiculturally, multilinguistically, multigenerationally, and socioeconomically diverse populations should analyze these data to identify access and use gaps for eHealth resources by seniors separately from the broader population. Most importantly, access to and use of eHealth resources should be monitored not only for the full senior population or the segment already known to be going online, but also by social determinants such as race/ethnicity, older age, low educational attainment, and low income. Government health agencies and quality assurance organizations focused on senior health and health care should hold health care providers and systems accountable for demonstrating that all patients are satisfied with the ease of communicating with their health care providers and the health care systems, their ability to get health and health care–related information and advice, and their ability to access reduced-cost services and products, regardless of whether they are able to go online.

Further research is needed to explore the extent to which age group and race/ethnic eHealth disparities affect patient-provider communication, use of patient education and disease management resources, and ultimately, health outcomes in different settings. Research is also needed to develop and evaluate the impact of improvements in the design of websites, patient portals, online patient education resources, self-monitoring tools, and eHealth devices that access Internet-based health resources aimed at reducing the physical, cognitive, psychomotor, emotional, and financial barriers that currently inhibit many seniors from using online resources for health-related purposes. Finally, more research is needed to develop and test interventions targeting seniors that aim to increase use of patient portals, eHealth devices, and other eHealth resources, including eHealth literacy programs, multimodal methods of providing website-specific training and support, and making home Internet more accessible to those on limited incomes.

## Appendix

Multimedia Appendix 1 Cell denominators for Tables 1 and 2.

Multimedia Appendix 2 Survey questionnaire.

Multimedia Appendix 3
Tables 1-9 showing exact P values for significance tests.

Multimedia Appendix 4 Cell denominators for Tables 8 and 9.

## Abbreviations

KPNC: Kaiser Permanente Northern California
non-LEP: non–limited English proficient
