# Supplementary material for: Differences in Access to and Preferences for Using Patient Portals and Other eHealth Technologies Based on Race, Ethnicity, and Age: A Database and Survey Study of Seniors in a Large Health Plan
Source: J Med Internet Res. 2016 Mar 4;18(3):e50. doi: 10.2196/jmir.5105 (PMC4799429; doi:10.2196/jmir.5105)
Supplement: Multimedia Appendix 1 [file jmir_v18i3e50_app1.pdf]

Cell denominators for Tables 1 and 2 (kp.org patient portal registration and utilization in 2013)

	Category						
	**1**	**2**	**3**	**4**	**5**	**6**	**7**
**Non-Hispanic white**							
**65–69**	42,956	37,067	36,957	32,438	33,600	29,668	23,754
**70–74**	80,354	66,397	70,530	59,356	65,255	55,130	49,572
**75–79**	60,255	45,471	50,748	39,285	49,695	38,437	41,426
**Black**							
**65–69**	3,986	2,442	3,385	2,152	3,158	2,042	2,929
**70–74**	7,325	4,059	6,302	3,600	5,940	3,417	5,643
**75–79**	5,587	2,642	4,588	2,248	4,487	2,195	4,434
**Latino**							
**65–69**	2,923	1,958	2,517	1,748	2,312	1,620	1,908
**70–74**	5,250	3,345	4,607	3,006	4,309	2,831	3,668
**75–79**	4,236	2,452	3,593	2,122	3,530	2,075	3,179
**Filipino**							
**65–69**	3,049	1,994	594	1,767	2,447	1,659	2,298
**70–74**	5,354	3,273	4,682	2,936	4,453	2,831	4,196
**75–79**	3,493	1,930	2,950	1,686	2,914	1,667	2,835
**Chinese**							
**65–69**	1,461	1,258	1,315	1,144	1,089	952	812
**70–74**	2,650	2,218	2,390	2,021	2,122	1,799	1,723
**75–79**	2,203	1,665	19,46	1,507	1,843	1,424	1,552

Categories:

1: All in race/ethnicity × age group

2: All in race/ethnicity × age group that had a kp.org account by end of 2013.

3: Subgroup of race/ethnicity × age group that had ≥1 lab test in 2013

4: Subgroup of race/ethnicity × age group that had a kp.org account by end of 2013 and ≥1 lab test in 2013

5: Subgroup of race/ethnicity × age group that had ≥1 prescription refill in 2013

6: Subgroup of race/ethnicity × age group that had a kp.org account by end of 2013 and ≥1 prescription refill in 2013

7: Subgroup of race/ethnicity × age group that was in the health plan's diabetes, hypertension, and/or coronary artery disease chronic disease registry
