# Supplementary material for: Differences in Access to and Preferences for Using Patient Portals and Other eHealth Technologies Based on Race, Ethnicity, and Age: A Database and Survey Study of Seniors in a Large Health Plan
Source: J Med Internet Res. 2016 Mar 4;18(3):e50. doi: 10.2196/jmir.5105 (PMC4799429; doi:10.2196/jmir.5105)
Supplement: Multimedia Appendix 2 [file jmir_v18i3e50_app2.pdf]

KAISER PERMANENTE®

DIVISION OF RESEARCH
Northern California

MEMBER SURVEY:
HEALTHCARE USING NEW TECHNOLOGIES

CONFIDENTIAL

Name
Street Address
City Zip

STUDY ID: 1234

Do we have your correct information in case we need to reach you? Please print any CHANGES below.

Address: ______________________________

____

Daytime phone: (____) ______________________

Email address: ______________________________

We are doing this survey to learn about how seniors prefer to communicate with Kaiser Permanente and get health information.

- ***Even if you do NOT use a mobile phone, computer or the Internet*, we want you to fill out this questionnaire. It is *very important* that we hear from people who do not use mobile phones or computers and are not interested in using the Internet or email as well as those who do use them!**
- **This questionnaire should be filled out *ONLY* by or for the person named above.**
- **You do not have to be a current Kaiser Permanente member to participate.**
- **You will receive a $5 gift card when we receive your completed questionnaire.**

Your answers are absolutely confidential. Your name and Study ID are on the questionnaire so we can note that you returned the questionnaire and contact you, if needed, to make sure we understand your answers. If you have any questions about confidentiality, the purpose of the survey, or how to complete the survey, please call toll-free: **(800) XXX-XXXX (choose Member Health Surveys)** or email me at PIname@kp.org.

Please return your completed survey in the enclosed postage-paid envelope to:
Kaiser Permanente Division of Research, 2000 Broadway, Oakland, CA 94612 attn: XXX

THANK YOU FOR YOUR PARTICIPATION!

1. Do you have a mobile phone (a regular cell phone or smartphone such as an iPhone)?

☐ No ➔ **Do you plan to get a mobile phone in the future?** ☐ Yes ☐ No ***(Now go to 2)***

☐ Yes ➔ ***Please answer 1a-1c in the box below***

1a. Do you also have a land-line (non-mobile phone) at home?

☐ Yes ☐ No

1b. Are you able to send and receive text messages on your mobile phone?

☐ Yes, I can do this by myself
☐ Yes, but someone else helps me or does this for me
☐ No, I cannot send or receive text messages

1c. What kind of mobile phone do you have?

☐ Regular cell phone (cannot download apps to it, but may be able to use for email or texting)
☐ Smartphone (such as an iPhone, Android, Samsung Galaxy) that can also be used to get onto the Internet, to download and use music, podcasts, and apps

➔ **<u>If you have a smartphone</u>: do you use apps on your phone?** ☐ Yes ☐ No

2. Do you own or have easy access to the following types of computers? *(Check ALL that apply)*

☐ Desktop computer ☐ Laptop computer ☐ Netbook ☐ Tablet (e.g., iPad, Samsung Galaxy)

3. Can you use the Internet to get information from websites or to communicate with others?

☐ No, I do not use the Internet ➔ ***Go to 4***
☐ Yes, I can do this by myself
☐ Yes, but someone else helps me or does this for me } ***Please answer 3a-3c in the box below***

3a. How often do you <u>usually</u> use the Internet (go on the web)?

☐ At least once a day ☐ A few times a week ☐ Once a week ☐ Less than once a week

3b. Where do you use the Internet? *(Check ALL that apply)*

☐ My home ☐ Library ☐ At work ☐ At a neighbor's/family member's home ☐ Other:_______

3c. What devices do you (or your helper) usually use to get information from websites? *(Check ALL that apply)*

☐ Computer, laptop, or netbook
☐ Tablet (e.g., iPad, Kindle, Nook)
☐ Web TV
☐ Smartphone (e.g., iPhone, Samsung Galaxy)
☐ Cell phone
☐ Other: ________________________________

4. Are you able to send and receive email messages?

☐ No, I do not use email ➔ ***Go to 5***
☐ Yes, I can do this by myself
☐ Yes, but someone else helps me or does this for me } ***Please answer 4a-4c in the box below***

4a. If you can receive email, how often do you usually check your email?

☐ At least once a day ☐ A few times a week ☐ Once a week ☐ Less than once a week

4b. Do you have your own email address or do you share an email address?

☐ My own email address ☐ Share with someone else ☐ Someone else receives my email

4c. What devices do you (or your email helper) usually use to check and send email?

☐ Computer/laptop ☐ Tablet (e.g., iPad) ☐ Smartphone ☐ Cell phone ☐ Other:_________

5. Are you able to send secure messages to your doctor or others through the kp.org website?

- ☐ No, I never do this ➔ ***Go to 6***
- ☐ Yes, I can do this by myself ➔ ***Go to 6***
- ☐ Someone helps me do this or does this for me ➔ ***Please answer 5a-5b in the box below***

5a. How is the person who usually helps you send secure messages related to you?

☐ Spouse/partner ☐ Son or daughter ☐ Other relative ☐ Friend ☐ Other:___________

5b. Does this person sign onto kp.org using <u>your</u> kp.org user ID and password or do they use <u>their own</u> kp.org user ID and password?

☐ My sign-on information ☐ Their own sign-on information ☐ I don't know

6. How do you prefer to communicate with your doctor <u>outside of the clinic</u> about <u>non-urgent</u> medical needs (such as asking follow-up questions after a visit or getting an opinion)?
(Check ALL methods <u>you use</u>, then put a ★ by the <u>one you most prefer</u> to use)

- ☐ Leave a **phone message** and get a return call
- ☐ Send a **secure message** through the kp.org website
- ☐ Send a **regular email message** (not through the kp.org website -- *not recommended*)
- ☐ Other *(describe)*:____

7. If your doctor thought it was <u>not necessary</u> for you to be seen in person, would you be willing to have:

	<u>No</u>	<u>Yes</u>	<u>Maybe</u>
**a. A <u>telephone appointment</u> if the co-pay was less than an office visit?** . . .	☐	☐	☐
**b. A <u>video visit</u> using your computer or smartphone so you and your doctor can see each other while you are talking?**	☐	☐	☐

8. Do you take one or more prescription medicines on an ongoing basis (e.g., for high blood pressure, diabetes, high cholesterol, heart problems, asthma, chronic pain, etc.)?

- ☐ No ➔ ***Go to 9***
- ☐ Yes ➔ ***Please answer 8a-8c in the box below***

8a. How do you usually prefer to <u>place orders</u> to <u>refill</u> your prescription medicines?
(Check ALL methods you use, then put a ★ by the <u>one you most prefer</u> to use)

- ☐ **Go to the pharmacy** and place the refill order in person
- ☐ Place the refill order **over the phone**
- ☐ Place the refill order **using the kp.org website**
- ☐ **Someone else** (son, daughter, spouse, etc.) **usually orders** my prescription refills for me
- ☐ Other *(describe)*:____

8b. How do you usually prefer to <u>receive</u> your prescription medicines?
(Check ALL methods you use, then put a ★ by the <u>one you most prefer</u> to use)

- ☐ **Go to the pharmacy** and pay for the medicine in person
- ☐ Have **someone else goes to the pharmacy to pick** them up for me
- ☐ Have the medicine **mailed to my home**

8c. If you don't use mail order or online prescription re-ordering services, why not?
(Check ALL reasons that apply and add others)

- ☐ I **don't have a credit card** to use to pay for mail order or online prescriptions
- ☐ I **prefer not to have my credit card number on file** with Kaiser Permanente
- ☐ I am concerned about the **medication being stolen from my mail box**
- ☐ I just **prefer to pick up** my medications at the pharmacy
- ☐ Other:____

9. When you have a lab test, how do you usually prefer to get the test result?
(Check ALL methods you use, then put a ★ by the one you most prefer to use)

- ☐ Have someone from Kaiser Permanente **call me** with the result
- ☐ Look up the result on **kp.org myself**
- ☐ Have **someone else** look up the result on kp.org for me
- ☐ Have my doctor send me a **secure message** with the result (requires signing in to kp.org)
- ☐ Get a **letter** in the mail

10. How would you be willing to receive health newsletters like our *Partners in Health* member newsletter or newsletters about specific health topics?
(Check ALL methods you would be willing to use, then put a ★ by the one you most prefer)

- ☐ Get a **print newsletter by regular mail**
- ☐ Get an **email newsletter** (the email text is the newsletter)
- ☐ Get an **email with a pdf attachment** to click on to read or print out the newsletter
- ☐ Get an **email with a link** to click on to read the newsletter on a website

11. How would you be willing to receive information from Kaiser Permanente or Medicare about your health care benefits or other information related to your health care?
(Check ALL methods you would be willing to use, then put a ★ by the one you most prefer)

- ☐ Get **print information by regular mail**
- ☐ Get an **email** containing the information
- ☐ Get an **email with a link** you need to click on to get to a website with the information
- ☐ Get an **email with a pdf attachment** to click on to read or print out the newsletter
- ☐ By a **phone message** from a computer system (also known as a Robo call)

12. How would you be willing to receive reminders about scheduled appointments or that you are due for lab tests, screening procedures, or flu shots?
(Check ALL methods you would be willing to use, then put a ★ by the one you most prefer)

- ☐ By letter or postcard sent by **regular mail**
- ☐ By **secure message** (requires signing onto kp.org to be able to read it)
- ☐ By **regular email** (no need to sign into kp.org to read the email, but you might need to sign into kp.org to get more detailed instructions or information)
- ☐ By a **phone message** from a computer system that may require you to enter your Health Plan number to hear the full message or respond
- ☐ Using the Kaiser Permanente **Preventive Care App** (for smartphone or tablet)

13. How would you be willing to complete yearly health assessment questionnaires? (These ask you to rate your health, ability to do things, and smoking, exercise, etc.)
(Check ALL methods you would be willing to use, then put a ★ by the one you most prefer)

- ☐ **Paper** (print) questionnaire
- ☐ **By phone using the phone keypad** to enter answers to questions read by a nice taped voice
- ☐ **Online** (web-based) questionnaire on the kp.org secure website
- ☐ **Using a touch screen tablet** or touch screen computer at the medical facility
- ☐ **Have someone ask** me the questions over the phone or in person

14. How would you be willing to complete health history questionnaires (such as intake forms for medical procedures)?
(*Check ALL methods you would be willing to use, then put a ★ by the one you most prefer*)

- ☐ **Paper** (print) questionnaire
- ☐ **By phone using the phone keypad** to enter answers to questions read by a nice taped voice
- ☐ **Online** (web-based) questionnaire on the kp.org secure website
- ☐ **Using a touch screen tablet** or touch screen computer at the medical facility
- ☐ **Have someone ask** me the questions over the phone or in person

15. How would you be willing to complete questionnaires for a <u>survey or research study</u>?
(Check ALL methods you would be <u>willing to use</u>, then put a ★ by the <u>one you most prefer</u>)

- ☐ **Paper** (print) questionnaire
- ☐ **By phone using the phone keypad** to enter answers to questions read by a nice taped voice
- ☐ **Online** (web-based) questionnaire on a secure website
- ☐ **Using a touch screen tablet** or touch screen computer at the medical facility/research site
- ☐ **Have someone ask** me the questions over the phone or in person

16. In which of the following ways would you like to get health information and advice in addition to directly from your doctor/clinicians? *(Mark YES for ways you'd like to use)*

Yes	I would like to:
☐	Have **phone appointments with** a Kaiser Permanente **Health Coach**
☐	Get help from a Kaiser Permanente **Health Coach by email**
☐	Watch a **DVD** using a computer, TV, or DVD player at home
☐	Watch an **online video** on the kp.org or another website like YouTube
☐	Watch a **video sent over the Internet** to a TV, DVD player, or computer by Netflix/other
☐	Use an **interactive DVD or CD-ROM on a computer** (answer questions, choose options)
☐	Watch live or recorded **online webinar talks/presentations** on a website like kp.org
☐	Use **online interactive programs** (read information, answer questions, get feedback)
☐	Listen to an **audio podcast** or other audio program on a computer, iPod or MP3 player
☐	Read about **health topics online** on a website like kp.org or WebMD
☐	Use a **health-related app** on a smartphone or tablet (e.g,, iPad.)
☐	Join an **online chat room or community** for members with similar health problems

17. Which of the following can you do on your own or with a little help? *(Mark YES if you can do)*

Yes	I am able to:
☐	Watch a **DVD on a television or computer** using a DVD player
☐	Use an **interactive CD-ROM or DVD program** on a computer
☐	Listen to an **audio CD** on a CD player
☐	Listen to a **podcast or music** on an iPod, MP3 player, or computer
☐	**Download a podcast** or music from a website to play on an iPod, MP3 player, or computer
☐	Read **health information online** on a website like kp.org
☐	Watch and listen to **videos on a website** such as kp.org or YouTube
☐	Watch **movies and programs sent to a TV or computer** over the Internet (e.g., Netflix)
☐	Watch or listen to a **webinar** (live talk given on a website) using a computer or smartphone
☐	**Read books or other print material** on a tablet (e.g., iPad) or e-reader (e.g., Kindle, Nook)
☐	**Enter information on a touch screen device** (computer, tablet, mobile phone)
☐	**Use Skype/other video chat service** to see the person I am talking to

18. Could you do the following tasks alone or with some help? *(Mark ONE answer for each task)*

**Tasks:**	**Could do by myself**	**Could do with help**	**Could not do**
**a. Send a message to your doctor** through the Kaiser Permanente website if you had a question	☐	☐	☐
**b. Look up a test result** on the Kaiser Permanente website	☐	☐	☐
**c. Complete a short questionnaire or form** on a computer	☐	☐	☐
**d. Complete a long questionnaire like this** on a computer	☐	☐	☐
**e. Complete a questionnaire using a touch screen tablet** (such as an iPad) while sitting in a clinic waiting room	☐	☐	☐
**f. Answer questions about your health using your phone's keypad** (e.g., Enter 1 if 'Always', 2 if 'Sometimes', 3 if 'Never')	☐	☐	☐
**g. Go to a website** to get information or forms **using a URL** (website address such as kp.org) given orally or in a letter	☐	☐	☐
**h. Print** information or forms from a website	☐	☐	☐
**i. Print** an **email** message	☐	☐	☐

19. Have you (alone or with someone's help) done any of the following <u>in the past 12 months</u>?
(Check an answer for each task you did. If you did not do a task, leave it blank)

**I did this myself**	**Someone helped me do this**	**In the past 12 months I did this:**
☐	☐	**Searched for information on the Internet** about any type of product, service or problem using Google or some other search engine
☐	☐	**Got information about a health topic** from <u>any</u> website (kp.org or other site)
☐	☐	**Downloaded or printed a form** or other document from a website
☐	☐	**Downloaded an app** from a website (Apple app store, Google Play, etc.)
☐	☐	**Clicked on a website link** that opened a document or took you to another part of the website or another website
☐	☐	Filled out an **online form or questionnaire** and then submitted it
☐	☐	**Paid a bill or ordered** any product or service **online using a credit card**
☐	☐	**Paid a bill or ordered** any product or service **online using PayPal**
☐	☐	Sent an email to a doctor or other health care professional using **regular email**
☐	☐	**Used online chat** to get information or help from a company's website *(i.e., typed a question into a chat box and got an answer typed back in a chat box)*
☐	☐	Looked at someone's or a company's **Facebook page** online
☐	☐	Used **Facebook** to post a message or 'Like' something
☐	☐	Used **Skype**/other video chat to view the person I was talking to on screen
☐	☐	Used **Twitter**
☐	☐	Used a **health-related app** (e.g., to monitor blood pressure, sleep, calories or food eaten, exercise, etc.) on a smartphone or iPad/other tablet computer

20. Do you have a physical problem (e.g., vision, shakiness) or other problems that make it difficult for you to use a computer or the Internet?

- ☐ No problems
- ☐ Yes I have problems *(describe):* ____
- ☐ I don't use for other reasons (*describe*):____

____

21. If you don't have Internet service <u>at home</u>, is this because of the cost?

- ☐ I have Internet at home
- ☐ Yes, I don't have it because it costs too much
- ☐ No, I don't for other reasons *(describe)* :____

22. Which of the following would you like to learn how to do on the Kaiser Permanente website?
(Mark YES for each you would like to learn how to do or check that you are not interested below)

☐ I am **not interested** in learning how to use the Kaiser Permanente website ➔ ***Go to 24***

Yes	**I would like to learn how to**:
☐	**Sign onto the kp.org website** to use secure features (communicate with my doctor, etc.)
☐	**Send secure messages** (kp.org email) to communicate with doctors/other clinicians
☐	**View and print out after-visit summaries** and recommendations from my doctor
☐	**View results of lab tests** and other procedures online
☐	**Order prescription refills** online
☐	**View, schedule, change or cancel medical appointments** online
☐	**Enter information into online health questionnaires** and other forms
☐	**Find information** about symptoms, conditions, and medications on the kp.org website
☐	**Find and watch online health videos** or **download podcasts or apps** from the website
☐	**Download forms** such as advanced directives and permission to release health information
☐	Other:

23. What would be the best ways for us to help you learn how to use the features on the Kaiser Permanente website? *(Mark YES for each you might like to use and suggest others)*

Yes	**Type of help I would like:**
☐	A **print handbook** listing what is on the website and the steps to access these features
☐	A **pdf version of a handbook** emailed to me or that I could download
☐	An **online handbook/directory** on the website with searchable content and FAQ section
☐	A **video** to watch on the kp.org website demonstrating how to use the website's features
☐	**Workshops given at a library** or community center that teach how to use the website
☐	**Someone who can be called** toll-free when I need help using the website
☐	Someone who can **help using live online chat** while I am on the website
☐	Other:

24. In your opinion, has Kaiser Permanente's shift toward using its website made it easier or harder for you to do the following? *(Mark ONE answer for each task)*

**Tasks**:	**Much Easier**	**Somewhat Easier**	**About Same**	**Somewhat Harder**	**Much Harder**
**a. Get information about your health plan benefits and costs**	☐	☐	☐	☐	☐
**b. Communicate with your doctor**	☐	☐	☐	☐	☐
**c. Get results of lab tests**	☐	☐	☐	☐	☐
**d. Get information you want about health conditions and treatments**	☐	☐	☐	☐	☐
**e. Get health education to help you improve your health or reduce risks**	☐	☐	☐	☐	☐
**f. Manage your health care**	☐	☐	☐	☐	☐

25. Do you have any comments about using the Internet (web) and other new technologies for communicating with clinicians, completing questionnaires, and getting health information?

____

____

26. Do you have any comments about Kaiser Permanente's website (kp.org or kp.org/mydoctor) or suggestions for what could make it easier to use or more useful?

____

____

About You

27. What is your sex? ☐ Male ☐ Female ☐ Transgender *(describe):* _____________

28. What is your age? ☐ Under 65 ☐ 65-69 ☐ 70-74 ☐ 75-79 ☐ 80-85 ☐ Over 85

29. What describes your race and ethnicity? *(Check ALL that apply)*

☐ Caucasian/White of European descent
☐ African-American/Other Black
☐ Mexican or Central American ancestry
☐ Other Hispanic/Latino: ________________
☐ Filipino
☐ Chinese
☐ Southeast Asian (Vietnamese, Thai, etc.)
☐ Japanese
☐ South Asian (Indian, Pakistani, Afghan, etc.)
☐ Other Asian: __________________________
☐ Pacific Islander/Native Hawaiian
☐ Middle Eastern/North African
☐ Native American Indian/Alaska native
☐ Other:________________________________

30. What is the highest level of school you completed? *(Check only ONE answer)*

☐ 8th grade or less *(no high school)*
☐ 9th - 11th grade *(some high school)*
☐ High school graduate or GED
☐ Some college/Associate's Degree
☐ Bachelor's Degree *(BA, BS, etc.)*
☐ Graduate or professional degree

31. In general, would you say your health is:

☐ Excellent ☐ Very good ☐ Good ☐ Fair ☐ Poor

32. What $5 gift card would you like us to send you? *(Mark ALL you would accept)*

☐ Target ☐ Safeway ☐ Starbucks ☐ Do not send a gift card

THANK YOU!
