# Supplementary material for: Differences in Access to and Preferences for Using Patient Portals and Other eHealth Technologies Based on Race, Ethnicity, and Age: A Database and Survey Study of Seniors in a Large Health Plan
Source: J Med Internet Res. 2016 Mar 4;18(3):e50. doi: 10.2196/jmir.5105 (PMC4799429; doi:10.2196/jmir.5105)
Supplement: Multimedia Appendix 3 [file jmir_v18i3e50_app3.pdf]

Table 1. Registration for and use of the patient portal by age group and race/ethnicity[a]

Use of the patient portal in 2013	Age	All	Non-Hispanic white	Black	Latino	Filipino	Chinese
**Was registered to use the patient portal by end of 2013, %**							
	65-79	77.1	81.1	54.1[b]	62.5[b]	60.5[b]	81.4
	65-69	82.2	86.3	61.3[b]	67.0[b]	65.4[b]	86.1
	70-74	78.6[c]	82.6[c]	55.4[b,c]	63.7[d,e]	61.1[b,c]	83.7
	75-79	71.5[c]	75.5[c]	47.3[b,c]	57.9[b,c]	55.2[b,c]	75.6[b,c]
**Signed onto the patient portal ≥1 time in 2013 (if registered to use patient portal at least part of 2013), %**							
	65-79	80.5	82.2	65.9[b]	70.8[b]	68.9[b]	85.5[b]
	65-69	83.3	85.0	69.4[b]	75.6[b]	74.1[b]	87.5
	70-74	81.6[c]	83.5[c]	66.9[b]	71.8[d,e]	69.1[b,c]	86.2[b]
	75-79	76.4[c]	78.2[c]	61.3[b,c]	65.7[b,c]	63.3[b,c]	83.1[b,c]
**Used the patient portal to send a message to a doctor, view lab test results, order a prescription refill, or make an appointment ≥1 time in 2013, %**							
**All members**							
	65-79	59.5	64.2	32.9[b]	41.4[b]	38.8[b]	67.3[c]
	65-69	65.9	70.8	39.6[b]	47.7[b]	45.3[b]	72.7
	70-74	61.7[c]	66.6[c]	34.3[b,c]	42.8[b,c]	39.3[b,c]	70.1[c]
	75-79	51.9[c]	56.3[c]	26.3[b,c]	35.1[b,c]	32.3[b,c]	60.3[c,e]
**Members registered to use the patient portal for at least part of 2013**							
	65-79	77.1	79.1	60.9[b]	66.2[b]	64.1[b]	82.6[b]
	65-69	80.1	82.0	64.7[b]	71.2[b]	69.3[b]	84.4
	70-74	78.5[c]	80.6 [c]	62.0[b]	67.2[d,e]	64.3[b]	83.8[b]
	75-79	72.6[c]	74.6 [c]	55.7[b,c]	60.7[b,c]	58.5[b,c]	79.8[b,c]
**Sent a secure message through the patient portal ≥1 time in 2013, %**							
**All members**							
	65-79	46.3	50.8	23.3[b]	30.1[b]	26.1[b]	49.1
	65-69	52.3	56.9	29.4[b]	35.2[b]	31.9[b]	54.2
	70-74	48.1[c]	52.8[c]	24.1[b,c]	31.1[b,c]	26.5[b,c]	52.0
	75-79	39.7[c]	43.7[c]	17.9[b,c]	25.4[b,c]	20.4[b,c]	42.2[c]
**Members registered to use the patient portal for at least part of 2013**							
	65-79	60.1	62.6	43.1[b]	48.2[b]	43.1[b]	60.3[b]
	65-69	63.5	66.0	47.9[b]	52.5[b]	48.8[b]	63.0
	70-74	61.3[c]	63.9[c]	43.6[b,c]	48.8[b]	43.3[b,c]	62.1
	75-79	55.5[c]	57.9[c]	37.9[b,c]	44.0[b,c]	36.9[b,c]	55.8[c]
**Viewed lab test results using the patient portal ≥1 time in 2013, %**							
**All members who had ≥1 lab test in 2013**							
	65-79	62.8	68.0	33.9[b]	42.7[b]	40.0[b]	69.1
	65-69	69.2	74.5	40.5[b]	49.6[b]	47.8[b]	74.6
	70-74	64.6[c]	69.9[c]	35.0[b,c]	44.1[b,c]	40.3[b,c]	71.7
	75-79	55.6[c]	60.6[c]	27.6[b,c]	36.0[b,c]	32.7[b,c]	62.2[c]
**Members who had ≥1 lab test in 2013 and were registered to use the patient portal during at least part of 2013**							
	65-79	79.6	82.1	60.6[b]	66.5[b]	64.1[b]	83.6
	65-69	82.4	84.8	63.7[b]	71.4[b]	70.2[b]	85.7
	70-74	80.6[c]	83.0 [c]	61.3[b]	67.6[b,g]	64.3[b,c]	84.9
	75-79	75.7[c]	78.3 [c]	56.4[b,c]	60.9[b,c]	57.3[b,c]	80.3[c]
**Ordered a prescription refill using the patient portal ≥1 time in 2013, %**							
**All members who refilled ≥1 prescription in 2013**							
	65-79	35.0	38.6	16.5[b]	21.2[b]	18.5[b]	37.0
	65-69	42.1	46.5	21.2[b]	26.6[b]	22.9[b]	44.4
	70-74	36.5[c]	40.4[c]	17.5[b,c]	21.8[b,c]	18.1[b,c]	38.4
	75-79	28.1[c]	31.0[c]	11.9[b,c]	16.9[b,c]	15.5[b,c]	31.1[c]
**Members who refilled ≥1 prescription in 2013 and were registered to use the patient portal at least part of 2013**							
	65-79	44.3	46.6	29.3[b]	33.0[b]	29.7[b]	44.8
	65-69	49.9	52.6	32.9[b]	38.0[b]	33.8[b]	50.8
	70-74	45.4[c]	47.8[c]	30.5[b]	33.3[b,h]	28.7[b,c]	45.2[d]
	75-79	38.3[c]	40.0[c]	24.3[b,c]	28.8[b,c]	27.1[b,c]	40.3[c]

Table1 notes:
[a]Cell percentages represent use among adults in that age, race/ethnic, or age-race/ethnic subgroup. The denominator for cell percentages in the “All” column includes all non-Hispanic white, black, Latino, Filipino, and Chinese members in that age group. See Multimedia Appendix 1 for cell denominators. Due to the very large denominators for all cells, comparisons with *P* values ≥.015 are not reported. See Multimedia Appendix 3 for detailed *P* values.
[b]Significantly differs (*P*<.001) from non-Hispanic whites within same age group after controlling for sex.
[c]Significantly differs (*P*<.001) from 65-69 age group within All or within same race/ethnic group after controlling for sex.
[d]Significantly differs (*P*=.003) from 65-69 age group within this race/ethnic group after controlling for sex.
[e]Significnatly differs (*P*=.001) from non-Hispanic whites in same age group after controlling for sex.
[f]Significantly differs (*P*=.009) from non-Hispanic whites in same age group after controlling for sex.
[g]Significantly differs (*P*=.008) from 65-69 age group within same race/ethnic group after controlling for sex.
[h]Significantly differs (*P*=.002) from 65-69 age group within same race/ethnic group after controlling for sex.

Table 2. Differences by age cohort and race/ethnic group in use of the health plan’s patient portal in 2013 among patients ages 65-79 who have diabetes, hypertension, and/or coronary artery disease[a]

Use of the patient portal in 2013	Age	All	Non-Hispanic white	Black	Latino	Filipino	Chinese
**Was registered to use the patient portal by end of 2013,%**							
	65-79	76.9	81.5	55.2[c]	63.0[c]	61.9[c]	82.0
	65-69	82.2	86.8	63.5[c]	68.3[c]	67.3[c]	87.9
	70-74	78.5[c]	83.3[c]	56.2[b,c]	64.6[c,d]	62.7[b,c]	83.7[e]
	75-79	71.9[c]	76.5[c]	48.4[b,c]	57.9[b,c]	56.3[b,c]	77.0[c]
**Used the patient portal to send a secure message to a doctor, view lab test results, order a prescription refill, or make an appointment ≥1 time in 2013, %**							
	65-79	63.3	69.2	36.2[c]	44.5[c]	42.8[c]	71.7[f]
	65-69	70.3	76.7	44.1[c]	51.4[c]	50.5[c]	78.6
	70-74	65.4[c]	71.6[c]	37.2[b,c]	46.4[b,c]	43.2[b,c]	74.6[g,h]
	75-79	56.6[c]	61.9[c]	29.9[b,c]	38.2[b,c]	35.8[b,c]	64.9[c]
**Sent a secure message through the patient portal ≥1 time in 2013, %**							
	65-79	49.3	54.8	25.8[b]	32.6[b]	28.7[b]	52.8[f]
	65-69	56.0	62.2	32.5[b]	38.2[b]	35.5[b]	59.6
	70-74	51.2[c]	57.0[c]	26.6[b,c]	34.1[c,I]	29.2[b,c]	56.6
	75-79	43.0[c]	47.9[c]	20.4[b,c]	27.6[b,c]	22.4b[b,c]	45.1[c,J]

[a]Study population for this table is members who were in a health plan diabetes, hypertension, or coronary artery disease registry in 2013. Cell percentages represent use among adults in that age, race/ethnic, or age-race/ethnic subgroup. The denominator for cell percentages in the “All” column includes all non-Hispanic white, black, Latino, Filipino, and Chinese members in that age group. See Multimedia Appendix 1 for cell denominators. Due to the large denominators for all cells, comparisons with *P* values ≥.055 are not reported. See Multimedia Appendix 3 for detailed *P* values.
[b]Significantly differs (*P*<.001) from non-Hispanic white within same age group after controlling for sex.
[c]Significantly differs (*P*<.001) from 65-69 age group within All or within same race/ethnic group after controlling for sex.
[d]Significantly differs (*P*=.007) from 65-69 age group within same race/ethnic group after controlling for sex.
[e]Significantly differs (*P*=.005) from 65-69 age group within same race/ethnic group after controlling for sex.
[f]Significantly differs (*P*=.010) from non-Hispanic white within same age group after controlling for sex.
[g]Significantly differs (*P*=.023) from 65-69 age group within same race/ethnic group after controlling for sex.
[h]Significantly differs (*P*=.013) from non-Hispanic white within same age group after controlling for sex.
[i]Significantly differs (*P*=.004) from non-Hispanic white within same age group after controlling for sex.
[j]Significantly differs (*P*<.008) from non-Hispanic white within same age group after controlling for sex.

Table 3. Characteristics of survey respondents, after weighting, by age group and race/ethnicity[a]

	All, %	By Age Group, %			By Race/Ethnicity,%				
	65-79 (N=2602)	65-69 (N=841)	70-74 (N=878)	75-79 (N=883)	Non-Hispanic white (N=849)	Black (N=567)	Latino (N=653)	Filipino (N=219)	Chinese (N=314)
**Age group**									
65-69	23.5	n/a	n/a	n/a	23.4	23.6	23.6	25.6	23.1
70-74	43.7	n/a	n/a	n/a	43.8	43.3	42.3	45.0	42.0
75-79	32.8	n/a	n/a	n/a	32.8	33.1	34.1	29.4	34.9
**Sex**									
Women	54.1	53.8	53.9	54.5	53.8	56.9	54.8	57.1	48.2
Men	45.9	46.2	46.1	45.5	46.2	43.1	45.2	42.9	51.8
**Race/ethnicity**									
White non-Hispanic	79.4	79.0	79.6	79.5	n/a	n/a	n/a	n/a	n/a
Black	7.3	7.3	7.3	7.4	n/a	n/a	n/a	n/a	n/a
Hispanic/Latino	5.4	5.4	5.2	5.6	n/a	n/a	n/a	n/a	n/a
Filipino	5.2	5.6	5.3	4.6	n/a	n/a	n/a	n/a	n/a
Chinese	2.7	2.7	2.6	2.9	n/a	n/a	n/a	n/a	n/a
**Educational attainment**									
Non-high school graduate	5.0	3.2	3.1	8.9[b]	3.9	4.7	22.0[c]	4.7	4.1
High school graduate/GED[d]	21.3	14.5	19.9	28.1	21.0	25.2	31.0	14.1	14.3
Some college	23.8	33.4	36.1	30.8	34.2	45.0	27.8	22.7	24.6
College graduate	39.9	48.9	40.9[e]	32.2[b]	40.9	25.0[c]	19.2[c]	58.4[c]	57.0[c]
**Household income in US$ in 2010 [d]**									
≤25,000	17.7	11.9	18.7[b]	26.7[b]	15.8	26.1[c]	22.3[c]	29.3[c]	16.8
25,001-35,000	12.6	10.0	13.2	16.5	11.9	16.3	17.5	17.5	8.7
35,001-80,000	42.0	42.9	42.6	39.6	42.1	39.7	43.9	43.9	41.3
>80,000	27.7	35.2	25.5[b]	17.2[b]	30.2	17.8[c]	16.3[c]	16.3[c]	33.3
**Self-rated health**									
Very good or excellent	43.8	49.7	46.8	35.6[b]	48.3	21.2[c]	28.0[c]	25.9 [c]	38.9[g]
Good	38.1	34.8	38.2	40.2	35.9	48.8	39.9	49.7	44.8
Fair or poor	18.1	15.5	15.0	24.2[b]	15.8	30.0[c]	32.1[c]	24.4[h]	16.3
History of diabetes, hypertension, coronary artery disease, heart failure, or stroke[i]	71.7	62.1	70.3[j]	80.6[b]	69.0	87.4[c]	77.8[c]	86.5[c]	70.5
Takes medication for ≥1 chronic condition	90.5	87.2	90.3	93.3[k]	89.9	95.4[c]	90.7	94.9[l]	86.8

[a]Cell percentages are based on weighted data for everyone in that age or race/ethnic group. Ns at top of columns are the unweighted number of respondents in that group. *P* values ≥.055 are not reported. See Multimedia Appendix 3 for detailed *P* values.
[b]Significantly differs (*P*<.001) from 65-69 age group after controlling for race/ethnicity and sex.
[c]Significantly differs (*P*<.001) from non-Hispanic white after controlling for age group and sex.
[d]GED = General Educational Development (credential indicating that individual has met high school level academic skills)
[e]Significantly differs (*P*=.020) from 65-69 age group after controlling for race/ethnicity and sex.
[f]Based on estimates from a 2011 health survey of the same health plan membership. A household income ≤$35,000 qualifies an individual for income-subsidized, low income housing.
[g]Significantly differs (*P*=.008) from non-Hispanic white after controlling for age group and sex.
[h]Significantly differs (*P*=.004) from non-Hispanic white after controlling for age group and sex.
[i]In ≥ 1 of the health plan's chronic disease registries for these conditions
[j]Significantly differs (*P*=.012) from 65-69 age group after controlling for race/ethnicity and sex.
[k]Significantly differs (*P*<.01) from 65-69 age group after controlling for race/ethnicity and sex.
[l]Significantly differs (*P*<.05) from non-Hispanic white after controlling for age group and sex.

Table 4. Seniors' access to digital devices, Internet, and email, by age group and race/ethnicity[a]

	All	By Age Group			By Race/Ethnicity				
	65-79 (N=2602)	65-69 (n=841)	70-74 (n=878)	75-79 (n=883)	Non-Hispanic white (n=849)	Black (n=567)	Latino (n=653)	Filipino (n=219)	Chinese (n=314)
**Has access to a mobile phone (cellular phone or smartphone),%**	81.0	88.4	84.3	71.2[b]	82.2	82.8	72.6[c]	70.2[c]	77.0
Has a smartphone	31.2	43.4	33.9[d]	18.7[b]	32.8	30.5	22.0[c]	19.6[c]	26.6[e]
Able to receive text messages	47.2	61.5	51.4[f]	31.4[b]	47.4	53.6[g]	41.0[h]	45.1	54.9
If has a mobile phone	60.5	71.2	63.1[i]	46.5[b]	60.5	59.6	68.4[j]	59.9	67.0
**Owns or has easy access to a computer, laptop, netbook, or tablet,%**	81.5	91.5	82.5[b]	73.1[b]	85.3	70.7[c]	63.0[c]	57.5[c]	82.8
Desktop, laptop, or netbook	79.5	90.4	80.0[b]	71.2[b]	83.5	69.1[c]	61.1[c]	53.1[c]	79.8
Tablet	25.1	34.3	27.4[k]	15.6[b]	27.1	16.0[c]	12.6[c]	20.1[l]	28.3
Has home Internet	83.8	91.3	85.5[d]	76.2[b]	87.4	71.9[g]	68.4[c]	61.0[c]	84.8
**Able to use the Internet, %**									
Able to use on own or with help	79.4	88.9	81.5[b]	68.7[b]	83.9	64.4[c]	58.2[c]	53.3[c]	79.2
Uses on own	69.4	80.7	70.8[b]	59.4[b]	74.4	51.8[c]	48.0[c]	39.3[c]	69.4
Uses with help or proxy uses	10.0	8.2	10.7	10.3	9.5	12.6	10.2	14.0	9.8
If uses the Internet, how frequently goes online	(n=1886)	(n=707)	(n=637)	(n=542)	(n=714)	(n=390)	(n=410)	(n=125)	(n=247)
Daily	64.2	70.0	64.3	58.8[m]	66.2	51.1[c]	53.7[c]	47.2[c]	66.0
≤1x/wk	16.6	16.7	16.7	18.8	15.1	28.3[c]	25.8[c]	26.2[n]	14.4
**Able to use email, %**	(N=2594)	(n=839)	(n=876)	(n=879)	(n=848)	(n=565)	(n=650)	(n=217)	(n=314)
Able to use by self or with help	79.3	86.1	81.2[o]	72.1[b]	83.4	63.2[c]	59.6[c]	58.5[c]	80.8
Uses on own	70.0	80.2	71.9[p]	60.3[b]	74.7	52.6[c]	49.1[c]	43.0[c]	72.6
Uses with help or proxy uses	9.3	5.9	9.3	11.8[m]	8.8	10.6	10.6	15.6[q]	8.2
Has an email address	76.2	82.4	78.4	68.6[b]	80.5	60.0[c]	57.9[c]	51.3[c]	76.3
Has own email address	63.8	68.5	66.3	57.2[b]	67.3	51.2[c]	48.2[c]	42.6[c]	65.7
Shares an email address (may also have own)	11.3	13.5	11.4	9.5	13.0	6.2[c]	6.9[c]	4.9[r]	8.2[s]
Uses someone else's email address	1.8	1.4	1.7	2.2	1.4	3.1[t]	3.6[u]	3.7[v]	2.4
If receives email, how frequently checks for email	(n=1866)	(n=682)	(n=630)	(n=554)	(n=699)	(n=377)	(n=420)	(n=124)	(n=246)
Daily	67.9	70.4	68.6	64.8	70.0	49.5[c]	59.5[c]	56.4[w]	70.5
≤1x/wk	13.8	12.4	12.2	17.4	12.3	24.6[c]	22.9[c]	22.2[x]	13.7

[a]Cell percentages based on weighted data for everyone in that age or race/ethnic group. Ns at top of columns are the unweighted number of respondents in that group except when analyses are restricted to a subset of that group. *P* values ≥.055 are not reported. See Multimedia Appendix 3 for detailed *P* values.

[b]Significantly differs (*P*<.001) from 65-69 age group after controlling for race/ethnicity and sex.

[c]Significantly differs (*P*<.001) from non-Hispanic white after controlling for age group and sex.

[d]Significantly differs (*P*=.005) from 65-69 age group after controlling for race/ethnicity and sex.

[e]Significantly differs (*P*=.053) from non-Hispanic white after controlling for age group and sex.

[f]Significantly differs (*P*=.004) from 65-69 age group after controlling for race/ethnicity and sex.

[g]Significantly differs (*P*=.033) from non-Hispanic white after controlling for age group and sex.

[h]Significantly differs (*P*=.023) from non-Hispanic white after controlling for age group and sex.

[i]Significantly differs (*P*=.030) from 65-69 age group after controlling for race/ethnicity and sex.

[j]Significantly differs (*P*=.006) from non-Hispanic white after controlling for age group and sex.

[k]Significantly differs (*P*=.031) from 65-69 age group after controlling for race/ethnicity and sex.

Table 4 notes (contd.):

[l]Significantly differs (*P* =.028) from non-Hispanic white after controlling for age group and sex.
[m]Significantly differs (*P* =.002) from 65-69 age group after controlling for race/ethnicity and sex.
[n]Significantly differs (*P* =.014) from non-Hispanic white after controlling for age group and sex.
[o]Significantly differs (*P* =.048) from 65-69 age group after controlling for race/ethnicity and sex.
[p]Significantly differs (*P* =.003) from 65-69 age group after controlling for race/ethnicity and sex.
[q]Significantly differs (*P* =.005) from non-Hispanic white after controlling for age group and sex.
[r]Significantly differs (*P* =.002) from non-Hispanic white after controlling for age group and sex.
[s]Significantly differs (*P* =.038) from non-Hispanic white after controlling for age group and sex.
[t]Significantly differs (*P* =.048) from non-Hispanic white after controlling for age group and sex.
[u]Significantly differs (*P* =.012) from non-Hispanic white after controlling for age group and sex.
[v]Significantly differs (*P* =.049) from Non-Hispanic white after controlling for age group and sex.
[w]Significantly differs (*P* =.008) from Non-Hispanic white after controlling for age group and sex.
[x]Significantly differs (*P* =.010) from Non-Hispanic white after controlling for age group and sex.

Table 5. Seniors' perceptions of their ability to perform health care–related tasks involving digital technology[a]

Task	All	By Age Group			By Race/Ethnicity				
	65–79 (N=2586)	65-69 (N=837)	70-74 (N=875)	75-79 (N=874)	Non-Hispanic white (N=847)	Black (N=562)	Latino (N=648)	Filipino (N=218)	Chinese (N=311)
**Send a message to doctor through the patient portal if had a question, %**									
Could do by self	64.2	76.1	66.3[b]	52.9[c]	68.6	47.2[d]	44.3[d]	40.9[d]	63.3
Could do by self or with help	79.7	88.2	81.7[b]	71.1[c]	82.9	67.2[d]	63.6[d]	66.1[d]	79.6
**Look up test result on the patient portal, %**									
Could do by self	64.5	76.4	66.8[b]	52.8[c]	69.0	45.7[d]	43.6[d]	40.2[d]	68.2
Could do by self or with help	78.4	87.3	80.5[b]	69.2[c]	81.9	63.8[d]	60.6[d]	61.2[d]	82.6
**Complete a short form or questionnaire on a computer, %**									
Could do by self	65.4	76.0	68.9[b]	53.2[c]	69.4	51.2[d]	45.3[d]	45.0[d]	67.2
Could do by self or with help	76.4	86.1	78.9[b]	66.3[c]	79.8	63.3[d]	59.3[d]	58.4[d]	79.7
**Complete a questionnaire using a touch screen tablet (such as an iPad) while sitting in a clinic waiting room, %**									
Could do by self	35.2	51.4	38.1[c]	20.0[c]	37.6	28.1[d]	23.0[d]	21.6[d]	34.3
Could do by self or with help	45.9	6358	49.3[c]	28.7[c]	47.7	40.6[e]	34.6[d]	36.1[f]	47.4
**Answer questions about your health using your phone's keypad (eg, Enter 1 if 'Always', 2 if 'Sometimes', 3 if 'Never'), %**									
Could do by self	52.1	62.5	55.0[g]	41.0[c]	54.6	49.4	37.8[d]	34.5[d]	50.3
Could do by self or with help	59.3	70.9	61.3[b]	48.5[c]	61.0	57.3	46.2[d]	50.9[e]	59.5
**Go to a website to get information or forms using a URL (website address) given orally or in a letter, %**									
Could do by self	50.9	66.9	52.1[c]	38.0[c]	55.2	37.9[d]	30.4[d]	24.3[d]	51.3
Could do by self or with help	60.2	75.3	61.8[c]	47.4[c]	63.3	49.6[d]	42.6[d]	42.9[d]	65.2
**Print information or forms from a website, %**									
Could do by self	60.6	72.0	63.2[b]	48.8[c]	65.3	46.2[d]	39.2[d]	30.8[d]	60.7
Could do by self or with help	70.4	81.7	72.3[c]	59.9[c]	74.0	58.1[d]	50.8[d]	50.8[d]	74.3

[a]Cell percentages are based on weighted data for everyone in the age or race/ethnic group. *P* values ≥.055 are not reported. See Multimedia Appendix 3 for detailed *P* values).Ns at top of columns are the unweighted number of respondents in that group.
[b]Significantly differs (*P*=.001) from 65-69 age group after controlling for race/ethnicity and sex.
[c]Significantly differs (*P*<.001) from 65-69 age group after controlling for race/ethnicity and sex.
[d]Significantly differs (*P*<.001) from non-Hispanic white after controlling for age group and sex.
[e]Significantly differs (*P*=.007) from 65-69 age group after controlling for race/ethnicity and sex.
[f]Significantly differs (*P*=.004) from 65-69 age group after controlling for race/ethnicity and sex.
[g]Significantly differs (*P*=.006) from 65-69 age group after controlling for race/ethnicity and sex.
[h]Significantly differs (*P*=.023) from 65-69 age group after controlling for race/ethnicity and sex.
[i]Significantly differs (*P*=.008) from 65-69 age group after controlling for race/ethnicity and sex.
[j]Significantly differs (*P*=.009) from non-Hispanic white after controlling for age group and sex.
[k]Significantly differs (*P*=.001) from non-Hispanic white after controlling for age group and sex.
[l]Significantly differs (*P*=.026) from 65-69 age group after controlling for race/ethnicity and sex.
[m]Significantly differs (*P*=.005) from 65-69 age group after controlling for race/ethnicity and sex.
[n]Significantly differs (*P*=.008) from non-Hispanic white after controlling for age group and sex.

Table 6. Methods used and preferred for performing tasks that could be done through the patient portal[a]

	All	By Age Group			By Race/Ethnicity				
	65-79	65-69	70-74	75-79	non-Hispanic white	Black	Latino	Filipino	Chinese
**Initiate non-urgent communications with doctors**	(N=2534)	(N=822)	(N=858)	(N=854)	(N=826)	(N=555)	(N=628)	(N=215)	(N=310)
**Send a secure message using the patient portal, %**									
Uses this method	58.2	70.0	58.7[b]	48.8[b]	63.6	33.8[c]	36.6[c]	32.2[c]	55.6[d]
Most prefers this method	51.8	63.9	53.8[e]	40.5[b]	57.7	25.2[c]	29.0[c]	25.0[c]	46.7[f]
**Send a message using regular email (discouraged), %**									
Uses this method	8.2	8.5	9.8	6.0	8.1	7.0	7.3	10.1	14.5[g]
Most prefers this method	4.8	3.5	6.4	3.5	4.9	3.3	4.4	3.3	7.5
**Leave phone message and get return call, %**									
Uses this method	53.7	48.0	50.0	62.6[b]	48.8	76.9[c]	71.3[c]	75.9[c]	58.0[h]
Most prefers this method	43.5	32.9	39.9[i]	56.0[b]	37.5	71.6[c]	67.0[c]	72.7[c]	45.8[j]
**Obtain results of lab tests**	(N=2594)	(N=838)	(N=874)	(N=882)	(N=847)	(N=566)	(N=649)	(N=219)	(N=313)
**Look up results online using the patient portal, %**									
Uses this method	54.4	64.9	55.4[k]	45.5[b]	58.8	31.1[c]	36.3	33.5[c]	63.6
Most prefers this method	38.9	47.3	41.2	30.1[b]	42.9	21.4[c]	21.4[c]	15.6[c]	45.7
**Result sent in a secure message using the patient portal, %**									
Uses this method	32.9	35.7	35.1	27.8[l]	34.2	25.0[c]	26.1[m]	32.7	28.2
Most prefers this method	19.1	19.6	20.4	17.2	20.4	11.1[c]	15.1[n]	19.6	10.7[c]
**Look up results online or get in secure message using the patient portal, %**									
Uses this method	66.6	74.4	69.3	57.5[b]	70.8	43.6[c]	48.4[c]	51.6[c]	71.4
Most prefers this method	57.9	66.8	61.2	47.3[b]	63.1	32.3[c]	36.3[c]	35.2[c]	56.5
**Get a letter in the mail with the result, %**									
Uses this method	51.8	48.6	50.3	56.0[o]	47.9	71.2[c]	66.3[c]	68.3[c]	53.6
Most prefers this method	35.6	28.7	33.1	44.0[b]	30.5	57.6[c]	54.0[c]	63.2[c]	40.4[p]
**Have someone from call with the result, %**									
Uses this method	18.4	16.7	17.1	21.4	17.7	27.9[c]	22.1	15.1	13.5
Most prefers this method	7.3	5.1	6.5	9.9[q]	7.1	10.5	11.0[r]	3.9	3.8
**Order prescription refills[s]**	(N=2258)	(N=715)	(N=764)	(N=779)	(N=731)	(N=521)	(N=561)	(N=187)	(N=258)
**Place order online using the patient portal, %**									
Uses this method	35.7	45.0	39.1	24.8[b]	39.7	20.0[c]	22.1[c]	12.8[c]	36.1
Most prefers this method	33.5	42.3	37.4	21.7[b]	37.2	16.3[c]	19.8[c]	12.2[c]	34.3
**Place order by phone, %**									
Uses this method	63.3	58.5	59.5	71.6[b]	61.4	70.8[m]	72.3[c]	72.7[t]	59.0
Most prefers this method	57.2	51.2	53.3	66.9[b]	55.9	61.8	64.5[p]	67.5[u]	52.8
**Place order in person at the pharmacy, %**									
Uses this method	20.6	18.9	19.8	23.0	17.3	37.0[c]	31.3[c]	32.6[c]	26.0[v]
Most prefers this method	9.5	6.5	9.7	11.4[w]	7.1	22.4[c]	16.1[c]	20.9[c]	12.9[x]
**Get reminders about appointments, immunizations, etc.**	(N=2586)	(N=835)	(N=871)	(N=880)	(N=843)	(N=565)	(N=646)	(N=218)	(N=314)
**Get a secure message using the patient portal, %**									
Uses this method	24.3	29.2	24.4	20.6[k]	26.6	16.0[c]	16.7[c]	9.5[c]	20.6
Most prefers this method	9.4	11.5	9.0	8.5	10.4	6.4[y]	6.0[y]	2.5[z]	8.9
**Get a regular email, %**									
Uses this method	44.8	54.1	48.9	32.8[b]	48.9	28.4[c]	26.2[c]	24.7[c]	44.7
Most prefers this method	29.2	33.6	35.1	18.3[b]	33.0	12.3[c]	13.3[c]	9.3[c]	25.4[aa]
**Get an automated phone message from a computer system, %**									
Uses this method	39.2	41.8	41.3	34.5	39.7	45.0	40.3	25.6[bb]	34.0
Most prefers this method	13.4	14.8	14.3	11.3	13.2	18.2[c]	18.7[j]	6.5[cc]	11.7
**Get a letter/postcard sent by regular mail, %**									
Uses this method	67.8	61.9	65.4	75.1[dd]	65.1	78.6[ee]	75.2[c]	85.1[c]	68.6
Most prefers this method	47.8	39.5	41.1	62.4[b]	43.2	63.5[c]	62.8[c]	80.5[c]	54.9[ff]
**Use the Kaiser Permanente preventive care app, %**									
Uses this method	5.2	6.4	6.7	2.4[e]	5.3	5.7	4.1	5.3	3.6
Most prefers this method	0.8	0.6	1.5	0.1	0.8	1.2	0.3	1.2	0.5

Table 6. Methods used and preferred for performing tasks that could be done through the patient portal[a]

	All	By Age Group			By Race/Ethnicity				
	65-79	65-69	70-74	75-79	non-Hispanic white	Black	Latino	Filipino	Chinese
**Complete health questionnaires**	(N=2570)	(N=832)	(N=862)	(N=876)	(N=839)	(N=560)	(N=643)	(N=216)	(N=312)
**Online questionnaire accessed via the patient portal, %**									
Uses this method	49.1	59.1	52.0[gg]	38.2[b]	54.5	28.3[c]	27.4[c]	21.1[c]	41.6[c]
Most prefers this method	35.1	42.3	39.3	24.2[b]	39.3	19.1[c]	18.6[c]	12.8[c]	27.8[c]
**Touchscreen tablet or computer at medical facility,%**									
Uses this method	7.4	11.5	7.9	3.6[b]	7.8	6.7	5.1[hh]	3.9[ii]	6.3
Most prefers this method	1.3	1.8	2.0	<0.1	1.5	<0.1	0.8	0.3	0.9
**Interactive Voice Response (IVR) questionnaire[jj], %**									
Uses this method	12.5	11.5	13.3	12.1	12.6	17.1[ii]	10.3	7.8	10.0
Most prefers this method	2.0	2.5	1.6	2.2	1.9	3.5	2.4	2.2	1.3
**Paper (print) questionnaire, %**									
Uses this method	77.2	70.4	76.1	83.5[b]	75.3	85.0[c]	83.1[c]	87.4[c]	79.3
Most prefers this method	56.6	50.1	52.4	67.0[b]	52.2	71.4[c]	71.1[c]	82.4[c]	66.6[c]
**Interviewer administered, %**									
Uses this method	18.5	13.2	18.8[gg]	21.7[kk]	18.3	24.0[y]	21.4	12.4	12.9
Most prefers this method	5.5	3.9	5.3	7.0	5.4	6.6	8.7[ll]	3.2	3.4

[a]Most preferred method restricted to people who indicated only one method or a most preferred method if >1 method was indicated. Cell percentages are based on weighted data for everyone in the age or race/ethnic group. Ns at top of columns are the unweighted number of respondents in that group. *P* values ≥.055 are not reported. See Multimedia Appendix 3 for detailed *P* values.

[b]Significantly differs (*P* < 001) from 65-69 age group after controlling for race/ethnicity and sex.

[c]Significantly differs (*P* <.001) from non-Hispanic white after controlling for age group and sex.

[d]Significantly differs (*P* =.021) from non-Hispanic white after controlling for age group and sex .

[e]Significantly differs (*P* =.005) from 65-69 age group after controlling for race/ethnicity and sex.

[f]Significantly differs (*P* =.004) from non-Hispanic white after controlling for age group and sex.

[g]Significantly differs (*P* =.002) from non-Hispanic white after controlling for age group and sex.

[h]Significantly differs (*P* =.010) from non-Hispanic white after controlling for age group and sex.

[i]Significantly differs (*P* =.045) from 65-69 age group after controlling for race/ethnicity and sex.

[j]Significantly differs (*P* =.024) from non-Hispanic white after controlling for age group and sex.

[k]Significantly differs (*P* =.004) from 65-69 age group after controlling for race/ethnicity and sex.

[l]Significantly differs (*P* =.013) from 65-69 age group after controlling for race/ethnicity and sex.

[m]Significantly differs (*P* =.001) from non-Hispanic white after controlling for age group and sex.

[n]Significantly differs (*P* =.022) from non-Hispanic white after controlling for age group and sex.

[o]Significantly differs (*P* =.028) from 65-69 age group after controlling for race/ethnicity and sex.

[p]Significantly differs (*P* =.007) from non-Hispanic white after controlling for age group and sex.

[q]Significantly differs (*P* =.023) from 65-69 age group after controlling for race/ethnicity and sex.

[r]Significantly differs (*P* = 037) from 65-69 age group after controlling for race/ethnicity and sex.

[s]Restricted to seniors who take medications for a chronic condition and do not rely totally on others to order their prescription refills.

[t]Significantly differs (*P* =.008) from non-Hispanic white after controlling for age group and sex.

[u]Significantly differs (*P* =.011) from non-Hispanic white after controlling for age group and sex.

[v]Significantly differs (*P* =.005) from non-Hispanic white after controlling for age group and sex.

[w]Significantly differs (*P* =.014) from 65-69 age group after controlling for race/ethnicity and sex.

[x]Significantly differs (*P* =.018) from non-Hispanic white after controlling for age group and sex.

[y]Significantly differs (*P* =.020) from non-Hispanic white after controlling for age group and sex.

[z]Significantly differs (*P* =.012) from non-Hispanic white after controlling for age group and sex.

[aa]Significantly differs (*P* =.033) from non-Hispanic white after controlling for age group and sex.

[bb]Significantly differs (*P* =.027) from 65-69 age group after controlling for race/ethnicity and sex.

[cc]Significantly differs (*P* =.048) from non-Hispanic white after controlling for age group and sex.

[dd]Significantly differs (*P* =.003) from non-Hispanic white after controlling for age group and sex.

[ee]Significantly differs (*P* =.030) from 65-69 age group after controlling for race/ethnicity and sex.

[ff]Significantly differs (*P* =.041) from non-Hispanic white after controlling for age group and sex.

[gg]Significantly differs (*P* =.029) from non-Hispanic white after controlling for age group and sex.

[hh]"By phone using the phone keypad to enter answers to questions read by a nice taped voice."

[ii]Significantly differs (*P* =.002) from 65-69 age group after controlling for race/ethnicity and sex.

[jj]Significantly differs (*P* =.033) from non-Hispanic white after controlling for age group and sex.

Table 7. Methods seniors are willing to use and would prefer for receiving newsletters and benefits information[a]

	All	By Age Group			By Race/Ethnicity				
	65–79	65–69	70–74	75–79	Non-Hispanic white	Black	Latino	Filipino	Chinese
**Get information about benefits or other topics related to your health**	(N=2581)	(N=838)	(N=865)	(N=878)	(N=839)	(N=564)	(N=647)	(N=219)	(N=312)
**Get an email containing all information in the body of the email, %**									
Willing to use this method	38.6	41.9	42.5	31.1[b]	41.4	27.8[c]	27.7[c]	22.9[c]	37.5
Most prefers this method	17.8	17.6	22.1	12.2[d]	19.8	8.6[c]	10.7[c]	8.5[c]	14.5
**Get an email with pdf attachment, %**									
Willing to use this method	23.3	31.9	25.9	13.7[b]	25.6	15.9[c]	12.2[c]	12.5[c]	18.1[e]
Most prefers this method	8.9	10.6	10.5	5.6[f]	10.0	5.7[g]	3.5[c]	2.9[c]	6.7
**Get an email with a link to a website, %**									
Willing to use this method	22.8	34.6	22.7[b]	14.6[b]	25.1	13.8[c]	13.1[c]	13.3[c]	18.1[h]
Most prefers this method	7.7	12.9	6.9	5.1	8.8	2.9[c]	4.1[i]	1.2[c]	5.7
**Get the information by one or more of the above types of emails, %**									
Willing to use this method	50.9	59.5	55.0	39.3[b]	54.8	36.0[c]	34.0[c]	30.7[c]	47.9[j]
Most prefers this method	34.3	41.1	39.5	22.8[b]	38.6	17.3[c]	18.4[c]	12.7[c]	26.9[i]
**Get print information by regular mail, %**									
Willing to use this method	76.6	74.0	73.3	82.7[k]	74.4	87.1[c]	84.0[c]	87.2[c]	76.9
Most prefers this method	60.7	52.6	56.4	72.1[b]	57.3	73.3[c]	75.3[c]	78.8[c]	64.1[l]
**Get an automated phone message[l], %**									
Willing to use this method	9.4	9.6	8.8	9.9	9.1	13.8[h]	13.0[n]	4.4[o]	6.4
Most prefers this method	0.6	0.9	0.3	0.7	0.4	1.8[p]	2.5[q]	0.4	<0.1
**Get health newsletters[m]**	(N=2377)	(N=769)	(N=790)	(N=818)	(N=815)	(N=480)	(N=594)	(N=187)	(N=301)
Get an email containing the newsletter in the body of the email, %									
Willing to use this method	39.2	44.0	44.5	28.5[b]	42.8	25.9[c]	24.0[c]	21.2[c]	33.4[r]
Most prefers this method	21.6	22.6	25.5	16.0[s]	23.9	11.4[c]	13.3[c]	11.7[c]	17.3[t]
**Get an email with a pdf attachment, %**									
Willing to use this method	23.8	31.8	25.3[u]	16.0[b]	26.4	16.0[c]	12.4[c]	11.2[c]	15.5[c]
Most prefers this method	10.4	11.9	12.1	7.0[s]	11.8	6.7[v]	5.0[c]	1.4[c]	7.1[w]
**Get an email with a link to a website, %**									
Willing to use this method	24.0	32.2	25.2[x]	16.4[b]	26.3	14.9[c]	13.7[c]	14.2[c]	21.9
Most prefers this method	9.5	13.9	10.4	5.3[b]	10.5	5.5[q]	5.1[i]	4.8[y]	10.5
**Get the newsletter in ≥1 of email types, %**									
Willing to use this method	54.5	62.5	59.3	42.2[b]	59.3	37.2[c]	34.4[c]	30.0[c]	48.2[l]
Most prefers this method	38.4	44.0	44.2	26.6[b]	42.7	21.2[c]	22.1[c]	16.5[c]	32.0[l]
**Get a print newsletter by regular mail, %**									
Willing to use this method	65.9	62.0	60.5	75.8[b]	62.7	80.3[c]	77.8[c]	80.8[c]	68.1
Most prefers this method	58.7	51.9	52.3	71.7[b]	54.1	76.4[c]	76.6[c]	82.2[c]	65.1[q]

[a]Most preferred method restricted to people who indicated only one method or a most preferred method if >1 method was indicated. Cell percentages are based on weighted data for everyone in the age or race/ethnic group. Ns at top of columns are the unweighted number of respondents in that group. *P* values ≥.055 are not reported. See Multimedia Appendix 3 for detailed *P* values.

[b]Significantly differs (*P* <.001) from 65-69 age group after controlling for race/ethnicity and sex.

[c]Significantly differs (*P* <.001) from WhiteNH after controlling for age group and sex.

[d]Significantly differs (*P* =.039) from 65-69 age group after controlling for race/ethnicity and sex.

[e]Significantly differs (*P* =.007) from non-Hispanic white after controlling for age group and sex.

[f]Significantly differs (*P* =.009) from 65-69 age group after controlling for race/ethnicity and sex.

[g]Significantly differs (*P* =.009) from non-Hispanic white after controlling for age group and sex.

[h]Significantly differs (*P* = .015) from non-Hispanic white after controlling for age group and sex.

[i]Significantly differs (*P* =.001) from non-Hispanic white after controlling for age group and sex.

[j]Differs (*P* = .050) from non-Hispanic white after controlling for age group and sex.

[k]Significantly differs (*P* =.002) from 65-69 age group after controlling for race/ethnicity and sex.

[l]Significantly differs (*P* =.002) from non-Hispanic white after controlling for age group and sex.

[m]Restricted to people who completed the longer form of the questionnaire

[n]Significantly differs (*P* =.035) from non-Hispanic white after controlling for age group and sex.

Table 7 notes continued:

[o]Significantly differs (P =.024) from non-Hispanic white after controlling for age group and sex.
[p]Significantly differs (P =.026) from non-Hispanic white after controlling for age group and sex.
[q]Significantly differs (P = 003) from non-Hispanic white after controlling for age group and sex.
[r]Significantly differs (P =.013) from non-Hispanic white after controlling for age group and sex.
[s]Significantly differs (P =.022) from 65-69 age group after controlling for race/ethnicity and sex.
[t]Significantly differs (P =.040) from non-Hispanic white after controlling for age group and sex.
[u]Significantly differs (P = 040) from 65-69 age group after controlling for race/ethnicity and sex.
[v]Significantly differs (P =.005) from non-Hispanic white after controlling for age group and sex.
[w]Significantly differs (P =.023) from non-Hispanic white after controlling for age group and sex.
[x]Significantly differs (P =.027) from 65-69 age group after controlling for race/ethnicity and sex.
[y]Significantly differs (P =.020) from non-Hispanic white after controlling for age group and sex.

Table 8. Willingness to perform health care–related tasks online[a]

	All	By Age Group			By Race/Ethnicity				
Health care–related tasks	65-79	65-69	70-74	75-79	Non-Hispanic white	Black	Latino	Filipino	Chinese
**Currently communicates at least sometimes with doctor using secure messaging when not urgent, %**									
All	58.2	70.0	58.7[b]	48.8[b]	63.6	33.8[c]	36.6[c]	32.2[c]	55.6[d]
Those who can use the Internet	71.8	77.9	70.3[b]	68.5[b]	74.6	51.0[c]	58.8[c]	58.1[c]	68.9
**Currently views lab test results online at least sometimes, %**									
All	54.4	64.9	55.4[e]	45.5[b]	58.8	31.1[c]	36.3[c]	33.5[c]	63.6
Those who can use the Internet	67.1	72.9	66.3[b]	63.2	69.0	47.4[c]	57.9[c]	60.0[c]	75.3
**Currently orders prescription refills online at least sometimes[g] , %**									
All	35.7	45.0	39.1	24.8[b]	39.7	20.0[c]	22.1[c]	12.8[c]	36.1
Those who can use the Internet	44.4	50.2	47.8	34.2[b]	46.8	29.9[c]	36.3[c]	22.8[c]	43.4
**Willing to complete health questionnaires online, %**									
All	49.1	59.1	52.0[g]	38.2[b]	54.5	28.3[c]	27.4[c]	21.1[c]	41.6[c]
Those who can use the Internet	61.5	66.6	63.5	53.7[b]	64.7	43.7[c]	45.3[c]	39.4[c]	51.5[c]
**Willing to complete health questionnaires in the clinic using a tablet or touchscreen computer, %**									
All	7.4	11.5	7.9	3.6[b]	7.8	6.7	5.1[h]	3.9[i]	6.3
Those who can use the Internet	9.1	13.0	9.4	5.1[b]	9.3	9.5	7.8	5.6	7.9
**Willing to read health information online at health plan or other website, %**									
All	35.4	42.4	38.4	26.4[b]	39.1	22.4[c]	20.5[c]	15.9[c]	28.3[j]
Those who can use the Internet	44.3	47.7	46.5	37.6[b]	46.4	34.0[c]	33.6[c]	29.8[c]	34.9[c]
**Willing to watch health videos online at health plan website or another website like YouTube, %**									
All	24.5	27.4	27.9	18.0[k]	26.7	17.5[c]	15.2[c]	11.6[c]	24.3
Those who can use the Internet	30.5	30.7	33.4	25.7	31.4	26.8	24.9[c]	20.2[c]	30.3
**Willing to consider (“yes” or “maybe”) a video visit with doctor instead of an office visit, %**									
All	33.8	44.6	36.6[k]	22.0[l]	36.5	24.8[m]	20.6[m]	19.3[m]	31.6
Those who can use the Internet	41.7	49.5	44.8	29.5[b]	42.8	37.7	32.8[m]	34.3[n]	36.0
**Willing to get health care–related information by email (in body of email, pdf attachment, or link), %**									
All	50.9	59.5	55.0	39.3[b]	54.8	36.0[c]	34.0[c]	30.7[c]	47.9
Those who can use the Internet	63.5	68.1	67.8	53.5[b]	65.1	56.3[c]	55.5[c]	52.9[c]	58.1

[a]Ability to use the Internet was assigned based on a “Yes” answer to the question “Can you use the Internet to get information from websites or to communicate with others?” Most senior Internet users were able go online on their own, but some indicated needing help or someone to go online for them. Ability to use email was assigned using the same type of question and responses. Cell percentages are based on weighted data for everyone in that age or race/ethnic group. Because percentages are based on responses to different questions, unweighted cell Ns vary. Most cell Ns can be ascertained from earlier tables, and they are also provided in Multimedia Appendix 4.

P values ≥.055 are not reported. See Multimedia Appendix 3 for detailed *P* values.

[b] Significantly differs (*P* < .001) from 65-69 age group after controlling for race/ethnicity and sex.

[c] Significantly differs (*P* < .001) from WhiteNH after controlling for age group and sex.

[d] Significantly differs (*P* = .021) from WhiteNH after controlling for age group and sex.

[e] Significantly differs (*P* = .004) from 65-69 age group after controlling for race/ethnicity and sex.

[f] Restricted to seniors who take medications for a chronic condition and do not rely totally on others to order their prescription refills.

[g] Significantly differs (*P* = .030) from WhiteNH after controlling for age group and sex.

[h] Significantly differs (*P* = .036) from WhiteNH after controlling for age group and sex.

[i] Significantly differs (*P* = .045) from WhiteNH after controlling for age group and sex.

[j] Significantly differs (*P* = .002) from WhiteNH after controlling for age group and sex.

[k] Significantly differs (*P* = .002) from WhiteNH after controlling for age group and sex.

[l] Significantly differs (*P* = .019) from 65-69 age group after controlling for race/ethnicity and sex.

[m] Significantly differs (*P* = .001) from WhiteNH after controlling for age group and sex.

[n] Significantly differs (*P* = .048) from WhiteNH after controlling for age group and sex.

Table 9. Seniors' opinions on the effect of technology on ease of health care communication and education[a]

	All	By Age Group		By Race/Ethnicity				
Health care–related tasks	65-79	65-74	75-79	Non-Hispanic white	Black	Latino	Filipino	Chinese
**Get information about your health plan benefits and costs, %**								
Easier	46.8	50.2	38.9[b]	46.1	48.0	47.2	53.7	50.5
Harder	16.8	13.8	23.7[c]	15.0	20.9[d]	23.4[e]	29.8[f]	21.1[g]
**Communicate with your doctor, %**								
Easier	73.3	76.5	65.9[h]	76.1	58.2[g]	63.2[g]	61.4[g]	69.3
Harder	11.8	9.6	17.0[h]	10.1	16.7[i]	18.7[g]	24.4[g]	14.6
**Ability to get lab test results, %**								
Easier	74.8	77.5	68.6[j]	77.2	60.0[g]	65.6[g]	64.9[k]	74.8
Harder	11.1	9.1	15.7[j]	9.5	16.6[e]	17.1[g]	21.0[g]	14.2
**Get information you want about health conditions and treatments, %**								
Easier	59.5	62.6	52.4[l]	60.3	53.3[m]	57.3	56.4	62.0
Harder	12.9	10.6	18.2[h]	11.0	18.0[n]	18.4[k]	26.8[g]	18.3[o]
**Get health education to help you improve your health or reduce risks, %**								
Easier	57.6	61.5	48.2[h]	58.3	52.5	54.3	56.6	57.8
Harder	12.4	10.1	17.8[b]	10.5	16.8[p]	20.0[g]	25.6[g]	17.1[q]
**Manage your health care, %**								
Easier	61.7	66.2	50.8[h]	63.2	53.5[i]	56.5[r]	54.4[s]	61.7
Harder	12.1	10.0	17.3[j]	10.5	15.5[t]	19.0[g]	23.9[g]	15.4

[a]Seniors were asked whether the health plan's shift toward using its website and patient portal has made it easier or harder for them to obtain information and communicate with their doctors. Analyses were restricted to people who expressed an opinion (including that there had been no change) about their ability to perform this task. Cell percentages are based on weighted data for everyone in the age or race/ethnic group. Because people did not indicate opinions about all tasks, unweighted cell Ns vary; they are provided in Multimedia Appendix 4. *P* values ≥.055 are not reported. See Multimedia Appendix 3 for detailed *P* values.

[b] Significantly differs (*P* = .001) from 65–74 age group after controlling for race/ethnicity and sex.

[c] Significantly differs (*P* = .024) from 65–74 age group after controlling for race/ethnicity and sex .

[d] Significantly differs (*P* = .001) from WhiteNH after controlling for age group and sex.

[e] Significantly differs (*P* = .050) from WhiteNH after controlling for age group and sex.

[f] Significantly differs (*P* < .001) from 65-74 age group after controlling for race/ethnicity and sex.

[g] Significantly differs (*P* <. 001) from WhiteNH after controlling for age group and sex.

[h] Significantly differs (*P* < .001) from 65-74 age group after controlling for race/ethnicity and sex.

[i] Significantly differs (*P* = .004) from WhiteNH after controlling for age group and sex.

[j] Significantly differs (*P* = .002) from 65-74 age group after controlling for race/ethnicity and sex.

[k] Significantly differs (*P* = .002) from WhiteNH after controlling for age group and sex.

[l] Significantly differs (*P* = .003) from 65-74 age group after controlling for race/ethnicity and sex.

[m] Significantly differs (*P* = .032) from WhiteNH after controlling for age group and sex.

[n] Significantly differs (*P* = .003) from WhiteNH after controlling for age group and sex.

[o] Significantly differs (*P* = .011) from WhiteNH after controlling for age group and sex.

[p] Significantly differs (*P* = .006) from WhiteNH after controlling for age group and sex.

[q] Significantly differs (*P* = .023) from WhiteNH after controlling for age group and sex.

[r] Significantly differs (*P* = .053) from WhiteNH after controlling for age group and sex.

[s] Significantly differs (*P* = .036) from WhiteNH after controlling for age group and sex.

[t] Significantly differs (*P* = .034) from WhiteNH after controlling for age group and sex.
