# Supplementary material for: Differences in Access to and Preferences for Using Patient Portals and Other eHealth Technologies Based on Race, Ethnicity, and Age: A Database and Survey Study of Seniors in a Large Health Plan
Source: J Med Internet Res. 2016 Mar 4;18(3):e50. doi: 10.2196/jmir.5105 (PMC4799429; doi:10.2196/jmir.5105)
Supplement: Multimedia Appendix 4 [file jmir_v18i3e50_app4.pdf]

Unweighted cell Ns for Table 8 (Willingness to perform healthcare-related tasks online)

	All	By Age Group			By Race/Ethnicity				
**Healthcare-related tasks**	**65–79**	**65–69**	**70–74**	**75–79**	**non-Hispanic white**	**Black**	**Latino**	**Filipino**	**Chinese**
Currently communicates at least sometimes with doctor using secure messaging when not urgent									
All	2534	822	858	854	826	555	628	215	310
Those who can use the Internet	1916	719	645	552	715	401	416	132	252
Currently views lab test results online at least sometimes									
All	2594	838	874	882	847	566	649	219	313
Those who can use the Internet	1943	725	654	554	731	403	423	132	254
Currently orders prescription refills online at least sometimes [a]									
All	2258	715	764	779	731	521	561	187	258
Those who can use the Internet	1697	620	572	505	635	370	366	113	213
Willing to complete health questionnaires online									
All	2570	832	862	876	839	560	643	216	312
Those who can use the Internet	1929	720	646	563	724	399	421	131	254
Willing to complete health questionnaires in the clinic using a tablet or touchscreen computer									
All	2570	832	862	876	839	560	643	216	312
Those who can use the Internet	1929	720	646	563	724	399	421	131	254
Willing to read health information online at health plan or other website									
All	2602	841	878	883	849	567	653	219	314
Those who can use the Internet	1948	728	656	564	732	404	426	132	255
Willing to watch health videos online at health plan website or another website like YouTube									
All	2602	841	878	883	849	567	653	219	314
Those who can use the Internet	1948	728	656	564	732	404	426	132	255
Willing to consider (“yes” or “maybe”) an MD video visit instead of office visit									
All	2597	838	878	881	848	567	652	216	314
Those who can use the Internet	1944	725	656	563	731	404	424	130	255
Willing to get healthcare-related information by email (in body of email, pdf attachment, or link)									
All	2581	838	865	878	839	564	647	219	312
Those who can use email	1942	715	647	580	717	395	432	139	259

[a] Restricted to seniors who take medications for a chronic condition and do not rely totally on others to order their prescription refills

Unweighted cell Ns for Table 9 (Seniors' opinions on the effect of technology on ease of healthcare communication and education)[a]

Healthcare-related tasks	All	By Age Group		By Race/Ethnicity				
	65–79	65–74	75–79	non-Hispanic white	Black	Latino	Filipino	Chinese
Get information about your health plan benefits and costs	1975	1344	631	667	421	463	175	259
Communicate with your doctor	2146	1462	684	729	444	508	186	279
Ability to get lab test results	2143	1463	680	735	441	499	184	284
Get information you want about health conditions and treatments	2050	1399	651	688	430	481	177	274
Get health education to help you improve your health or reduce risks	1981	1356	625	668	416	457	173	267
Manage your healthcare	2016	1380	636	678	424	466	175	273

[a] Responses are restricted to people who expressed an opinion (including that there had been no change) about their ability to perform this task.
